# Allometric equations for integrating remote sensing imagery into forest monitoring programmes

**DOI:** 10.1111/gcb.13388

**Published:** 2016-07-06

**Authors:** Tommaso Jucker, John Caspersen, Jérôme Chave, Cécile Antin, Nicolas Barbier, Frans Bongers, Michele Dalponte, Karin Y. van Ewijk, David I. Forrester, Matthias Haeni, Steven I. Higgins, Robert J. Holdaway, Yoshiko Iida, Craig Lorimer, Peter L. Marshall, Stéphane Momo, Glenn R. Moncrieff, Pierre Ploton, Lourens Poorter, Kassim Abd Rahman, Michael Schlund, Bonaventure Sonké, Frank J. Sterck, Anna T. Trugman, Vladimir A. Usoltsev, Mark C. Vanderwel, Peter Waldner, Beatrice M. M. Wedeux, Christian Wirth, Hannsjörg Wöll, Murray Woods, Wenhua Xiang, Niklaus E. Zimmermann, David A. Coomes

**Affiliations:** ^1^ Forest Ecology and Conservation Group Department of Plant Sciences University of Cambridge Cambridge UK; ^2^ Faculty of Forestry University of Toronto 33 Willcocks Street Toronto ON M5S 3B3 Canada; ^3^ Swiss Federal Research Institute WSL Zürcherstrasse 111 Birmensdorf 8903 Switzerland; ^4^ Laboratoire Evolution et Diversité Biologique UMR5174, CNRS/Université Paul Sabatier Bâtiment 4R1 118 route de Narbonne Toulouse F‐31062 France; ^5^ Institut de Recherche pour le Développement UMR AMAP Montpellier France; ^6^ Institut Français de Pondichéry UMIFRE CNRS‐MAE 21 Puducherry India; ^7^ Forest Ecology and Forest Management Group Wageningen University PO Box 47 AA Wageningen 6700 the Netherlands; ^8^ Department of Sustainable Agro‐ecosystems and Bioresources Research and Innovation Centre Fondazione E. Mach, Via E. Mach 1 San Michele all'Adige 38010 Italy; ^9^ Department of Geography and Planning Queen's University Kingston ON Canada; ^10^ Chair of Silviculture Faculty of Environment and Natural Resources Freiburg University Tennenbacherstr. 4 Freiburg 79108 Germany; ^11^ Department of Botany University of Otago PO Box 56 Dunedin 9016 New Zealand; ^12^ Landcare Research PO Box 69040 Lincoln 7640 New Zealand; ^13^ Kyushu Research Center Forestry and Forest Products Research Institute Kumamoto 860‐0862 Japan; ^14^ Department of Forest and Wildlife Ecology University of Wisconsin‐Madison Madison WI 53706 USA; ^15^ Faculty of Forestry University of British Columbia 2424 Main Mall Vancouver BC V6T 1Z4 Canada; ^16^ Laboratoire de Botanique systématique et d'Ecologie Département des Sciences Biologiques Ecole Normale Supérieure Université de Yaoundé I Yaoundé Cameroon; ^17^ Fynbos Node South African Environmental Observation Network (SAEON) Centre for Biodiversity Conservation Kirstenbosch Gardens Private Bag X7, Rhodes Drive, Claremont Cape Town 7735 South Africa; ^18^ Forest Research Institute of Malaysia Kepong 52109 Selangor Malaysia; ^19^ Department of Earth Observation Friedrich‐Schiller University Loebdergraben 32 Jena 07743 Germany; ^20^ Program in Atmospheric and Oceanic Sciences Princeton University Princeton NJ 08544 USA; ^21^ Botanical Garden of the Russian Academy of Sciences (Ural branch) Russia and Ural State Forest Engineering University Yekaterinburg 620100 Russia; ^22^ Department of Biology University of Regina 3737 Wascana Pkwy Regina SK S4S 0A2 Canada; ^23^ Systematic Botany and Functional Biodiversity Institute of Biology University of Leipzig Leipzig Germany; ^24^ German Centre for Integrative Biodiversity Research (iDiv) Halle‐Jena‐Leipzig Leipzig Germany; ^25^ Conservation and Natural Resources Management Sommersbergseestr. 291 Bad Aussee A‐8990 Austria; ^26^ Ontario Ministry of Natural Resources North Bay ON P1A 4L7 Canada; ^27^ Faculty of Life Science and Technology Central South University of Forestry and Technology Changsha 410004 China

**Keywords:** aboveground biomass, airborne laser scanning, carbon mapping, crown architecture, height–diameter allometry, stem diameter distributions

## Abstract

Remote sensing is revolutionizing the way we study forests, and recent technological advances mean we are now able – for the first time – to identify and measure the crown dimensions of individual trees from airborne imagery. Yet to make full use of these data for quantifying forest carbon stocks and dynamics, a new generation of allometric tools which have tree height and crown size at their centre are needed. Here, we compile a global database of 108753 trees for which stem diameter, height and crown diameter have all been measured, including 2395 trees harvested to measure aboveground biomass. Using this database, we develop general allometric models for estimating both the diameter and aboveground biomass of trees from attributes which can be remotely sensed – specifically height and crown diameter. We show that tree height and crown diameter jointly quantify the aboveground biomass of individual trees and find that a single equation predicts stem diameter from these two variables across the world's forests. These new allometric models provide an intuitive way of integrating remote sensing imagery into large‐scale forest monitoring programmes and will be of key importance for parameterizing the next generation of dynamic vegetation models.

## Introduction

**Figure 1:**
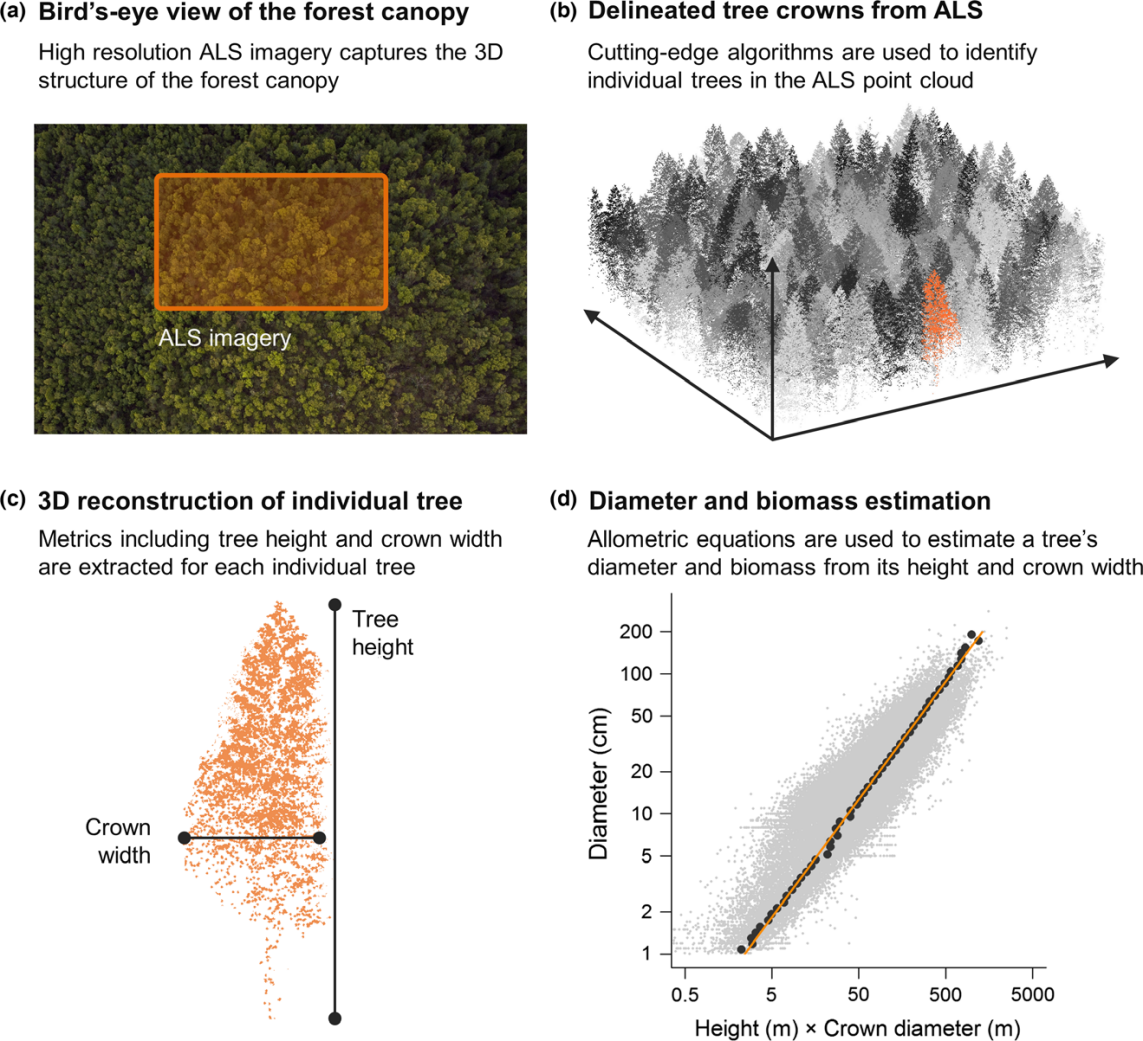
Schematic diagram illustrating how airborne laser scanning (ALS) imagery can be integrated into forest inventory programmes. State‐of‐the‐art algorithms that detect and measure individual tree crowns from ALS point clouds are combined with existing field data to estimate the diameter and aboveground biomass of remotely sensed trees.

Forests are a key component of the terrestrial carbon cycle (Beer *et al*., 2010; Pan *et al*., 2011), making forest conservation of critical importance for mitigating climate change (Agrawal *et al*., 2011). Yet effectively managing forests as carbon sinks is predicated on the assumption that carbon stocks can be quantified with accuracy across extensive and often remote areas. Traditionally, forest carbon stocks have been assessed by measuring the diameter (and sometimes height) of trees in permanent field plots and then using allometric equations to estimate biomass (Malhi *et al*., 2006; Pan *et al*., 2011; Anderson‐Teixeira *et al*., 2015). Recently, however, we have begun to see a move towards remote sensing as the primary tool for monitoring forest carbon (Saatchi *et al*., 2011; Baccini *et al*., 2012; Avitabile *et al*., 2016). Airborne laser scanning (ALS) is particularly promising in this regard (Asner & Mascaro, 2014; Asner *et al*., 2014), allowing the 3D structure of entire forest landscapes to be reconstructed in detail using high‐frequency laser scanners mounted on airplanes or unmanned aerial vehicles. Importantly, advances in both sensor technology and computation mean we are now able – for the first time – to reliably identify and measure the crown dimensions of individual trees using ALS (Yao *et al*., 2012; Duncanson *et al*., 2014; Shendryk *et al*., 2016), marking a fundamental shift in the way we census forests. To facilitate this transition, we aim to develop allometric equations for estimating a tree's diameter and aboveground biomass based on attributes which can be remotely sensed – namely tree height and crown diameter – enabling airborne imagery to be fully integrated into existing carbon monitoring programmes (Fig. 1).

While ALS opens the door to rapidly and accurately measuring the height and crown dimensions of millions of trees (Duncanson *et al*., 2015), it also poses the challenge of how best to use these data to estimate aboveground biomass. Current allometries rely on stem diameter as a key input for estimating biomass (e.g. Chave *et al*., 2014). But because diameters cannot be measured directly through ALS, new approaches that have tree height and crown dimensions at their centre are needed. We see two possible solutions for integrating tree‐level ALS data into biomass monitoring programmes: the first is to use tree height and crown dimensions to predict diameters, allowing biomass to be estimated using existing allometric equations (Dalponte & Coomes, 2016). The second is to develop equations that estimate biomass directly from tree height and crown size, thereby bypassing diameter altogether.

### Approach 1: estimating diameter

Theory based on the mechanical and hydraulic constraints to plant growth predicts that tree height (*H*, in m) should scale with diameter (*D*, in cm) following a power‐law relationship with an invariant scaling exponent of 2/3 (*H* ∝ *D* ^2/3^; West *et al*., 1999). This would suggest that measuring tree height should be sufficient for estimating diameter. However, growing evidence indicates that this is unlikely to be the case (Muller‐Landau *et al*., 2006): not only do *H–D* allometries vary considerably among and within species, as well as in relation to climate and stand structure (Banin *et al*., 2012; Lines *et al*., 2012; Hulshof *et al*., 2015; Jucker *et al*., 2015), but power‐law relationships also fail to adequately capture the asymptotic nature of height growth (Muller‐Landau *et al*., 2006; Banin *et al*., 2012; Feldpausch *et al*., 2012; Iida *et al*., 2012; Chave *et al*., 2014). Trees typically invest heavily in height growth when young to escape shaded understories – rapidly approaching their maximum height – but then continue to grow in diameter throughout their lives (King, 2005). This makes estimating the diameter of large trees challenging, as trees of similar height can have very different diameters – which is problematic given that large‐diameter trees hold most of the biomass (Slik *et al*., 2013; Bastin *et al*., 2015). In this context, information on crown size may prove key to accurately estimating a tree's diameter. While height growth tends to slow rapidly in large trees, lateral crown expansion does not, requiring a continued investment in stem growth on the tree's part to ensure structural stability and hydraulic function (Sterck & Bongers, 2001; King & Clark, 2011; Iida *et al*., 2012). As a result, crown width and stem diameter tend to be strongly coupled, even in large trees (Hemery *et al*., 2005).

### Approach 2: estimating aboveground biomass

Estimating the diameter of individual trees from remotely sensed data is an appealing prospect: not only would it provide a way to quantify biomass stocks, but would also allow other forest attributes of interest to be reconstructed with ease (e.g. stem diameter distributions). However, it also presents a challenge from the point of view of biomass estimation, as biomass allometries typically have diameter as a squared term in the equation (Zianis *et al*., 2005; Chave *et al*., 2014; Chojnacky *et al*., 2014), meaning that even small errors in diameter predictions can strongly influence the accuracy of biomass estimates. A better approach may therefore be to estimate a tree's aboveground biomass directly from crown architectural properties which can be measured from airborne imagery, without the need to first predict diameter. Specifically, both tree height (Hunter *et al*., 2013; Chave *et al*., 2014) and crown dimensions (Henry *et al*., 2010; Goodman *et al*., 2014; Ploton *et al*., 2016) are known to relate strongly to aboveground biomass, although it remains to be tested whether they can be used to accurately estimate biomass without needing to also account for stem diameter.

**Figure 2:**
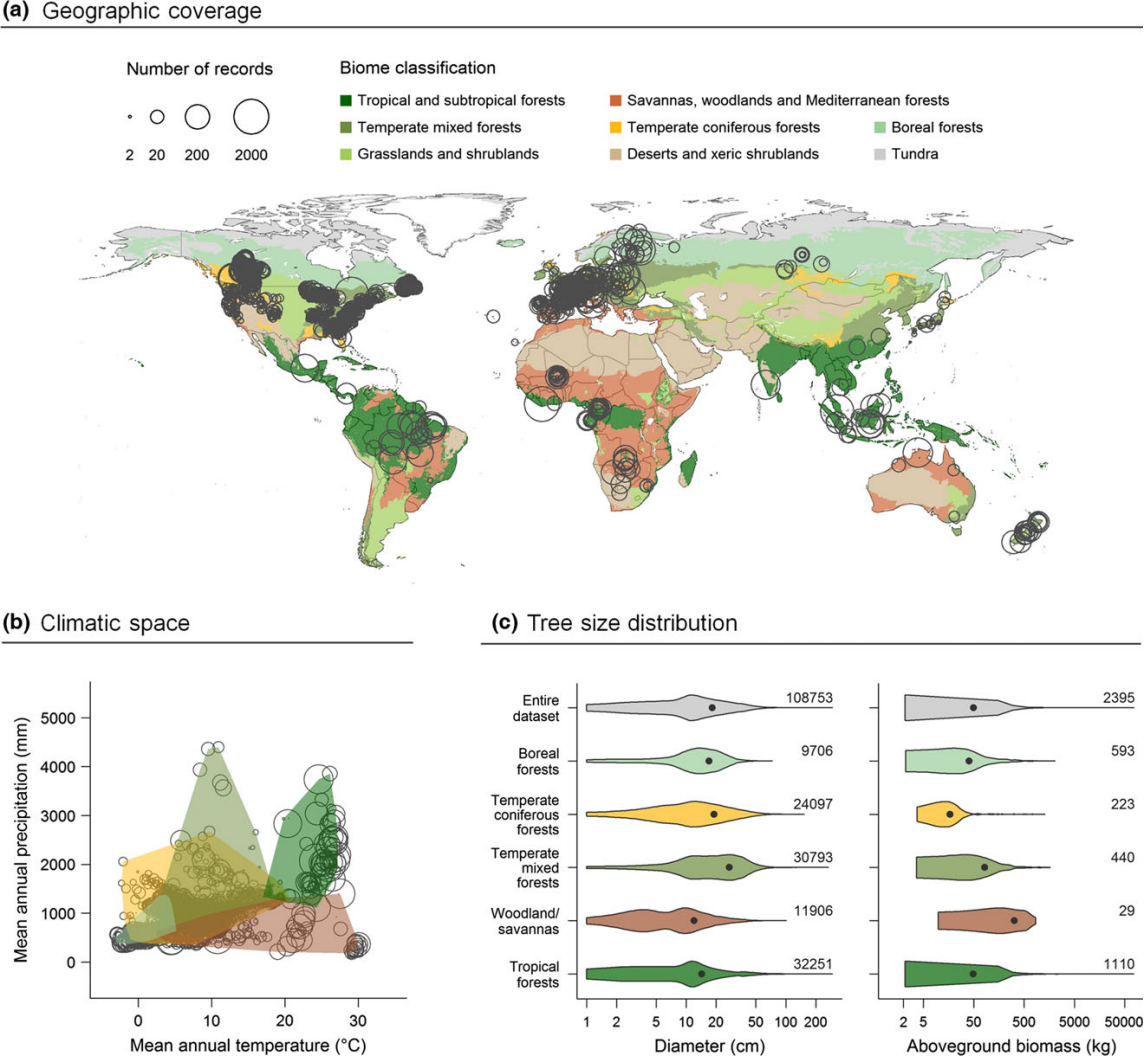
Overview of the allometric database. Panel (a) shows the geographic coverage of the database in relation to the world's biomes (map adapted from Olson *et al*., 2001). Circle size reflects the number of trees measured at each location (on a logarithmic scale). Panel (b) highlights differences in mean annual precipitation and temperature among forest types. Climate data were obtained from the WorldClim database (Hijmans *et al*., 2005), which consists of gridded annual mean values covering the period between 1950 and 2000 (data available from: http://www.worldclim.org/current). In (c) violin plots show the size distribution – in terms of diameter and aboveground biomass – of trees in the database. The number of records available for each forest type is displayed on the right.

Here we compile a global data set consisting of 108753 trees for which stem diameter, height and crown diameter have all been measured, including 2395 trees which have been harvested to measure aboveground biomass. The data set is representative of the world's major tree‐dominated biomes and spans a huge gradient in tree size (Fig. 2). We use these data to develop allometric equations that enable the precise and unbiased estimation of a tree's diameter and aboveground biomass based on its height and horizontal crown dimensions and use the following questions to guide our processes: (i) Can a tree's diameter be estimated accurately based on its height alone, or do we also need to account for its crown dimensions? (ii) Can a single universal equation be used to model diameter, or do different scaling relationships among forest types, biogeographic regions and tree functional types need to be accommodated for? (iii) Can a tree's aboveground biomass be estimated directly from its height and crown diameter, thereby eliminating the need to first predict its diameter?

## Materials and methods

### Allometric database

We compiled a global database of trees for which stem diameter (*D*, in cm), height (*H*, in m) and crown diameter (*CD*, in m) were all measured. Trees were selected for inclusion in the database based on the following criteria: (i) only trees with *D *≥* *1 cm and *H *≥* *1.3 m were considered; (ii) trees from managed plantations and agroforestry systems were excluded; (iii) trees known or presumed to be severely damaged were removed (e.g. broken stems or major branches; see Fig. S1); (iv) only trees whose geographic location was recorded were retained; and (v) from a taxonomic perspective trees had to, at a minimum, be identifiable as either angiosperms or gymnosperms (note that tree ferns and palms were excluded from the analysis). Our search yielded a total of 108753 trees which met the above requirements. For 2395 of these, total oven‐dry aboveground biomass (*AGB*, in kg) was additionally measured by harvesting and weighing trees. The database spans a large range of tree sizes (*D*: 1.0–293.0 cm; *H*: 1.3–72.5 m; *CD*: 0.1–41.0 m; *AGB*: 0.1–76063.5 kg), captures a wide spectrum of tree forms and functional types (1492 tree species from 127 families) and covers the major forest types and climatic conditions found in the world's forests (see Fig. 2 for an overview of the database). A full list of data sources and associated measurement protocols is provided in Appendix S1 of Supporting Information. The database is publicly available through figshare (https://dx.doi.org/10.6084/m9.figshare.3413539.v1), with data from the Alberta Permanent Sample Plots (https://www.agric.gov.ab.ca/app21/forestrypage) and the International Cooperative Programme on Air Pollution Effects on Forests (http://icp-forests.net/page/data-requests) archived separately and available upon request through the above links.

### Forest biome classification

Scaling relationships between *D*,* H* and *CD* are strongly influenced by climate (Lines *et al*., 2012; Hulshof *et al*., 2015), as well as varying among species (Poorter *et al*., 2006) and geographic regions (Banin *et al*., 2012). To capture this degree of variation – which we expect to be of key importance to accurately estimating both *D* and AGB – each tree in the database was assigned to one of five biome types based on its geographic location: boreal forests, temperate coniferous forests, temperate mixed forests, woodlands and savannas (which combines temperate and tropical savannas, as well as Mediterranean woodlands) or tropical and subtropical forests (biome classification follows Olson *et al*., 2001). In the same way, trees were also assigned to one of six biogeographic regions: Australasia, Afrotropics, Nearctic, Indo‐Malaya, Neotropics or Palearctic. Transitions among forest biomes reflect strong climatic gradients (Whittaker, 1975; Stephenson, 1998; Fig. 2b), whereas biogeographic realms define regions which share a common evolutionary history (Udvardy, 1975). Olson *et al*.'s (2001) map of the world's terrestrial ecoregions, which defines the geographic distribution of the world's major biome and biogeographic regions, is available for download from http://www.worldwildlife.org/publications/terrestrial-ecoregions-of-the-world.

### Approach 1: estimating diameter

#### Model development

To determine how to most accurately estimate a tree's diameter based on its crown architectural properties, we compared a set of regression models in which *D* was expressed as a function of either *H*,* CD* or the compound variable *H × CD* (which tests whether both height and crown size are needed to predict *D*). We chose to model the combined effect of *H* and *CD* using a compound variable (as opposed to including the two predictors separately in the model) to avoid issues with collinearity resulting from the nonindependence of *H* and *CD* (Dormann *et al*., 2013). Furthermore, preliminary analyses revealed that *H × CD* was as good (if not better) a predictor of *D* than a model with *H* and *CD* as separate explanatory variables (Table S2).

Typically, allometric equations are derived by fitting a linear regression directly to raw data (which in most cases have been log‐transformed). Yet this approach will tend to underestimate the slope of a bivariate line when the independent variable is measured with error (also known as regression dilution bias; Fuller, 1987; Warton *et al*., 2006). In the case of forest inventory data, this systematic bias is made worse by the inherently unbalanced size distribution of trees, as small stems – which vastly outnumber large ones – come to dominate the signal of the regression (Duncanson *et al*., 2015). As a solution to this problem, Duncanson *et al*. (2015) proposed fitting allometric models to binned data as opposed to raw values. Because this method reduces tree‐level variation in allometric attributes to a mean value, it has the drawback of inevitably underestimating the true uncertainty of the model. However, a preliminary analysis of the data revealed it to be the only approach able to adequately capture underlying allometric scaling relationships (see Appendix S2 for a detailed discussion). As a compromise, we therefore chose to adopt Duncanson *et al*.'s (2015) binning method to estimate allometric relationships, but also develop a framework for robustly quantifying and propagating model uncertainty when working with binned data (see ‘*Model uncertainty and error propagation’* section below).

We calculated the mean *H*,* CD* and *H × CD* for each of 50 stem diameter logarithmic bins of constant width (logarithmic binning was chosen to better capture the right‐skewed distribution of *D*). Linear log–log models were then fit to the binned data using least‐squares regression (as implemented in the r statistical software; R Core Development Team, 2013):

(1) $$\ln\left(D\right)=\alpha +\beta \ln\left(H\right)+\varepsilon$$

(2) $$\ln\left(D\right)=\alpha +\beta \ln\left(CD\right)+\varepsilon$$

(3) $$\ln\left(D\right)=\alpha +\beta \ln\left(H\times CD\right)+\varepsilon$$

where *α* and *β* are parameters to be estimated from the data and *ε* is an error term [which is assumed to be normally distributed, with a mean of zero and a standard deviation *σ*,* N*(0, *σ* ^2^).

Models 1–3 can be thought of as global allometric equations, as they assume that scaling relationships between *D*,* H* and *CD* are invariant across forest types, biogeographic regions and tree functional groups (e.g. angiosperms and gymnosperms). To determine the extent to which regional or group‐specific allometries improve the accuracy of *D* estimates compared to those of a global model, we used mixed‐effects models to develop two further equations. First, the relationship between *D* and the independent variable (e.g. *H × CD*) was allowed to vary among forest types nested within biogeographic regions (i.e. random intercept and slope model, where forest type and biogeographic region were treated as nested random effects). In the second model, the relationship between *D* and the independent variable was further allowed to vary among angiosperm and gymnosperm trees (i.e. separate *α* and *β* estimates were calculated for each functional group/forest type/biogeographic region combination). Note that to fit these models, the data binning processes was repeated and separate mean values of *H*,* CD* and *H × CD* were calculated for each combination of functional group, forest type and biogeographic region.

#### Generating predictions

Allometric models, such as those described above, can be used to estimate *D* for any tree whose *H* and *CD* are known. Using Model 3 as an example, predicted diameter values (*D*_pred_) are obtained as follows: *D*_pred_ = exp[*α* + *β*ln(*H* × *CD*) + *ɛ*]. Assuming *ε* is normally distributed [i.e. *N*(0, *σ* ^2^)], the mean of exp(ε) can be approximated by exp(*σ* ^2^/2), where *σ* ^2^ is the mean square error of the regression (Baskerville, 1972). An unbiased estimate of *D* can therefore be calculated using the following equation:

(4) $$D_{\mathrm{pred}}=\exp\left[\alpha +\beta \ln\left(H\times CD\right)\right]\times \exp\left[\sigma^{2}/2\right]$$

#### Model validation

To evaluate and compare the predictive accuracy of the different *D* models, we: (i) divided the database into a training set (90% of the data) and a validation set (remaining 10% of the data, used exclusively to evaluate model performance). Trees assigned to the validation data set were selected following a size‐stratified random sampling approach which aimed to capture the full range of *D* in the database; (ii) *D* models were fit to the training data set using the binning approach described above; (iii) fitted equations were used to predict *D* for all trees in the validation data set [as outlined in Eqn (4)]; and (iv) the predictive error of each model was quantified by comparing predicted and observed *D* values (*D*_pred_ and *D*_obs_, respectively) of trees in the validation data set (see below for a description of the model performance metrics used). Steps (i–iv) were repeated 100 times to avoid the randomization procedure in step (i) having an undue effect on the model evaluation process.

For each *D* model we calculated two measures of average error: the root mean square error (RMSE, in cm) and the relative systematic error (or bias, in %).

$$\mathrm{RMSE}=\sqrt{\frac{1}{N}\sum_{i=1}^{N}{\left(D_{\mathrm{obs}}-D_{\mathrm{pred}}\right)}^{2}}$$

$$\mathrm{Bias}=\frac{1}{N}\sum_{i=1}^{N}\left(\frac{D_{\mathrm{obs}}-D_{\mathrm{pred}}}{D_{\mathrm{obs}}}\right)\times 100$$

Additionally, a third model performance statistic was used to compare the predictive accuracy of the *D* models across functional groups (angiosperms and gymnosperms), forest types and biogeographic regions. Following the approach of Chave *et al*. (2014), we calculated the tree‐level coefficient of variation (CV) in *D* for trees of functional group *i*, growing in forest type *j* and in biogeographic region *k* as follows:

$${\mathrm{CV}}_{ijk}=\frac{{\mathrm{RMSE}}_{ijk}}{\frac{1}{N}\sum_{i=1}^{N}D_{\mathrm{obs}ijk}}$$

where RMSE_*ijk*_ is the RMSE of trees belonging to functional group *i*, growing in forest type *j* and in biogeographic region *k*, whereas the denominator corresponds to the mean observed *D* for this same group of trees. Standardizing the RMSE by the mean *D* is a necessary step to compare model errors across functional groups, forest types or biogeographic regions, as errors in *D* are strongly dependent on tree size (Colgan *et al*., 2013).

#### Model uncertainty and error propagation

As discussed previously, while data binning is well suited to estimating average allometric scaling relationships, it inevitably underestimates the true variability in these relationships among individual trees. Specifically, the data binning approach will tend to underestimate *σ* – the residual standard deviation – which makes quantifying and propagating uncertainty a challenge. In a linear modelling framework $\sigma =\sqrt{\frac{\sum {\left(y_{i}-{\hat{y}}_{i}\right)}^{2}}{n-2}}$, where *n* is the number of observations, *y*_*i*_ is the *i*th observation of the response variable, and ${\hat{y}}_{i}$ is the corresponding predicted value obtained from the model. The reason why data binning generally underestimates *σ* is that the difference between observed and predicted values (i.e. the residuals, $y_{i}-{\hat{y}}_{i}$) is calculated not for individual trees, but for mean values obtained by averaging across multiple trees. However, using an independent data set (the 10% of trees set aside for model validation), we can compare predicted and observed estimates of *D* generated for individual trees to get a much more realistic estimate of the true value of *σ* for a given model (which we refer to as *σ*_*v*_):

$$\sigma_{v}=\sqrt{\frac{\sum {\left(\ln\left(D_{\mathrm{obs}}\right)-\ln\left(D_{\mathrm{pred}}\right)\right)}^{2}}{n-2}}$$

Using this simple approach, we were able to generate realistic estimates of the predictive uncertainty of models fit using the data binning method (see Fig. S3). To enable users to robustly propagate uncertainty when using the equations developed here, we report *σ*_*v*_ values for all fitted models. Furthermore, in Appendix S5 we provide R code for replicating the entire analysis.

#### Scaling‐up from diameter to aboveground biomass

Approach 1 aims to predict *D* from crown attributes, with the idea that *D* estimates can then be fed into existing biomass equations. To quantify the extent to which replacing field‐measured *D* values with predicted ones influences the accuracy of *AGB* estimates, we used Chave *et al*.'s (2014) general biomass equation as a baseline. In Chave *et al*. (2014), AGB is expressed as the following function of *D*,* H* and wood density [*ρ*, in g cm^−3^; which we obtained from the global wood density database of Chave *et al*. (2009) and Zanne *et al*. (2009)]: AGB = 0.0673 × (*D* ^2^×*H*×*ρ*)^0.976^ × exp[0.357^2^/2]. Using this equation, we estimated AGB for trees in the database with a known biomass (i.e. trees that had been destructively harvested and weighed) using both field‐measured and predicted *D* values as inputs to the biomass model. Only trees with *D *≥* *5 cm were used for this purpose (*n *=* *1859 trees with field‐measured AGB), as trees smaller that this threshold contribute negligibly to forest carbon stocks and were not used to calibrate Chave *et al*.'s (2014) equation. By comparing observed AGB values with those predicted using Chave *et al*.'s (2014) equation, we were then able to determine whether the underlying *D* models described previously can be used to generate accurate biomass estimates. Additionally, this also allowed us to compare the predictive accuracy of approaches 1 and 2 – the latter of which aims to estimate AGB directly from *H* and *CD* (see following section).

### Approach 2: estimating aboveground biomass

Instead of estimating *D* first, a better approach to predicting the biomass of individual trees from crown architectural attributes might be to relate *AGB* directly to *H* and *CD*. To test this, we used data for trees with measured *AGB* to explore a number of alternative models relating *AGB* to *H* and/or *CD*. Preliminary analyses revealed the compound variable *H × CD* to be a far superior predictor of *AGB* than either *H* or *CD* alone. We therefore focus on the following log–log regression model of *AGB*:

(5) $$\ln\left(AGB\right)=\alpha +\beta \ln\left(H\times CD\right)+\varepsilon$$

Model development and validation followed the same steps described for Approach 1. As for previous equations, the model was fit to binned mean values of *H × CD* (as opposed to raw data). To allow a comparison with Approach 1, only trees with *D *≥* *5 cm were used to develop the model. We further tested whether modelling angiosperms (*n *=* *1069) and gymnosperms (*n *=* *790) separately would improve model accuracy, as these two functional groups differ strongly in crown architecture (Poorter *et al*., 2012; Hulshof *et al*., 2015) as well as wood density (Chave *et al*., 2009). Given the relatively small number of trees with measured *AGB* values, we did not explore the extent to which the relationship between *AGB* and *H × CD* varies among forest types or biogeographic regions. The predictive accuracy of Eqn (5) was compared against that of *AGB* models which include *D* as a predictor (i.e. Approach 1) on the basis of RMSE and bias.

## Results

### Approach 1: estimating diameter

**Figure 3:**
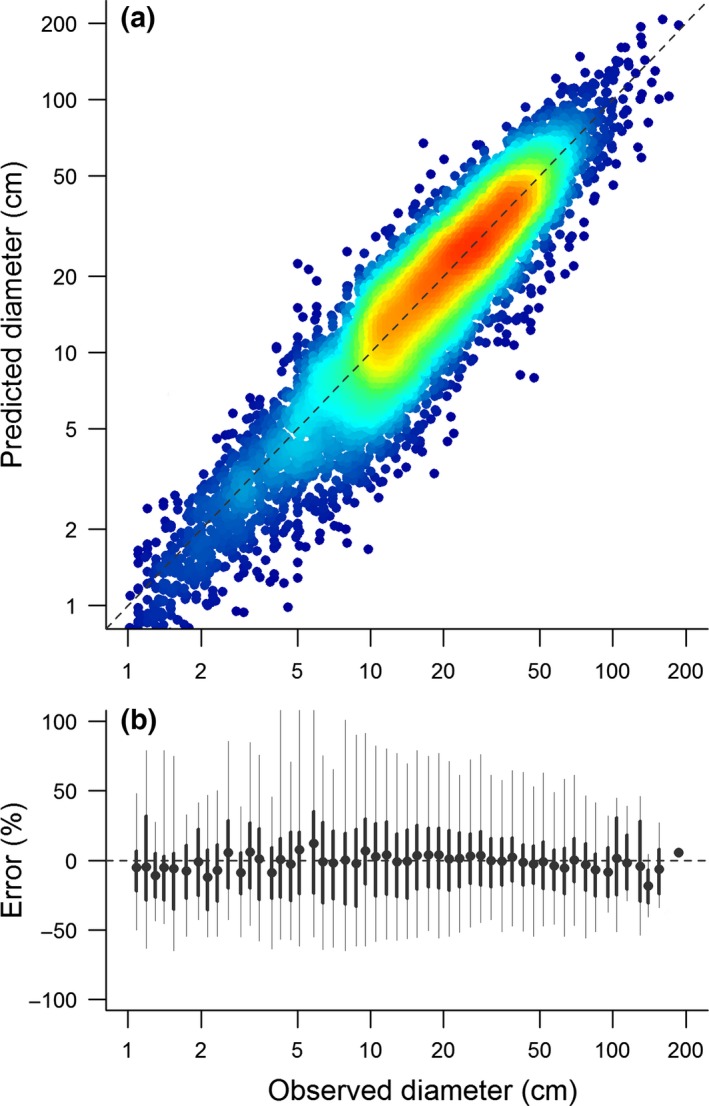
Goodness of fit for the global diameter model [i.e. Eqn (6) in the main text], tested on an independent random sample of the data corresponding to 10% of measured trees (*n *=* *10875). Panel (a) compares predicted and observed diameter values, with the dashed line corresponding to a 1 : 1 relationship. The density of overlapping points is represented by a colour gradient which ranges from blue (low point density) to red (high point density). Panel (b) reports the mean relative error (i.e. *D* = *α*(*H* × *CD*)^*β*^) for different diameter size classes, with the bars delimiting the interquartile range (thick lines) and 95% limits (thin lines) of the errors.

Of the candidate models we tested for estimating *D*, ones relying on *H* or *CD* alone as predictors of *D* proved unsuitable. Despite exhibiting relatively low RMSE (13.7 cm), a height‐only model tended to systematically overestimate *D* (bias = 24.7%). This occurred because *D–H* relationships were nonlinear on a log–log scale, as *H* tended to asymptote in large trees. As a result, a power‐law tended to overestimate *D* for small and medium‐sized trees, while severely underestimating that of large ones (Fig. S4). Conversely, a model with only *CD* as a predictor of *D* had higher RMSE (16.6 cm), but showed lower overall systematic bias (−4.5%). However, the average bias masks a tendency of the crown diameter‐only model to overestimate *D* for large trees, while underpredicting the size of smaller stems (Fig. S4). In contrast to the previous two models, *H × CD* proved a much better predictor of *D* (Fig. 3). The best‐fit global *D* model was

(6) $$D_{\mathrm{pred}}=0.557\times {\left(H\times CD\right)}^{0.809}\times \exp\left[0.056^{2}/2\right]$$

Equation (6) had both lower RMSE (9.7 cm) and average systematic bias (−1.2%) compared to models based on *H* or *CD* alone. Importantly, the model showed no evidence of over‐ or underpredicting *D* across a wide range of tree sizes (Fig. 3b). Using the independent validation data set, we estimated *σ*_*v*_ [i.e. the standard deviation of ln(*D*_obs_) – ln(*D*_pred_)] of the model to be 0.45.

**Figure 4:**
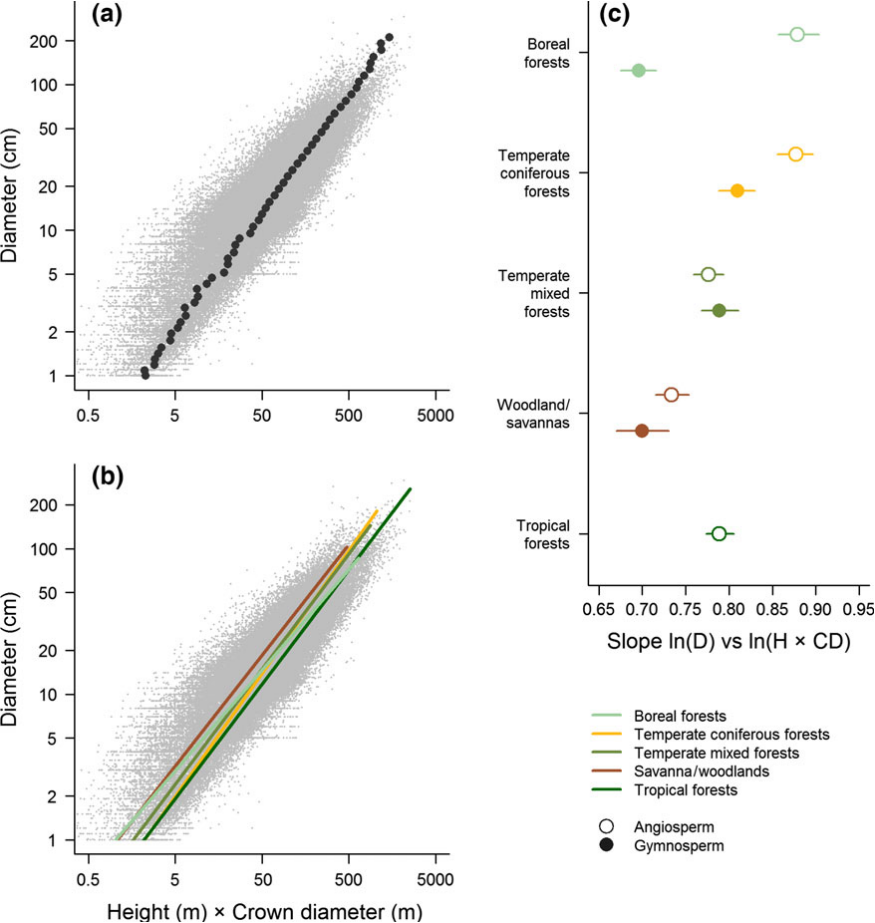
Relationship between stem diameter and the product of tree height and crown diameter (*H × CD*). Panel (a) shows the distribution – on a logarithmic scale – of the raw data (in grey) and of the mean *H × CD* values in each diameter size class (black circles). Panel (b) illustrates fitted relationships between diameter and *H × CD* for each forest type separately, while (c) reports the slopes of these relationships (± 95% confidence intervals) for angiosperms and gymnosperms separately.

**Figure 5:**
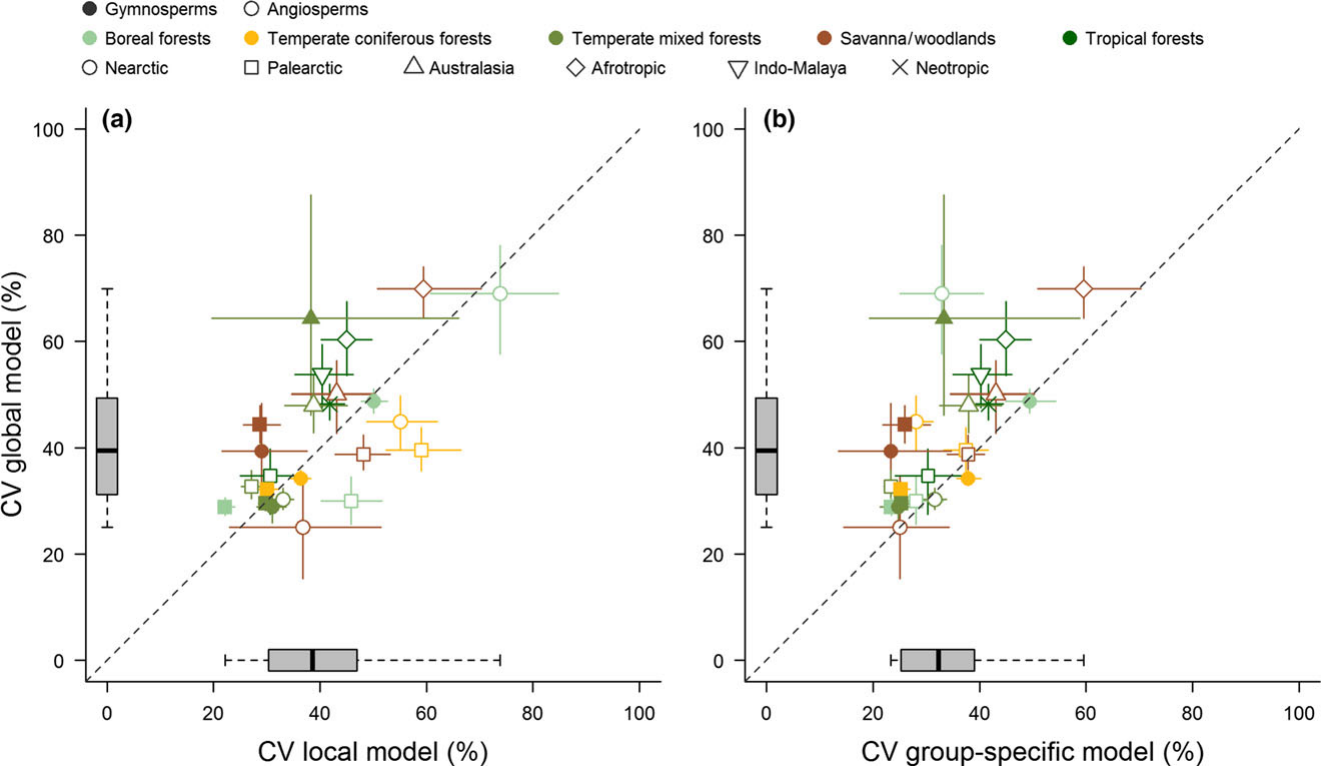
Comparison of model performance between the global diameter model [i.e. Eqn (6) in the main text] and (a) a model that allows scaling relationships to vary among forest types and biogeographic regions, and (b) one where angiosperms and gymnosperms are also modelled separately. The coefficient of variation (CV) of the absolute errors (±95% range across 100 simulations) is reported for angiosperms (open symbols) and gymnosperms (closed symbols) according to forest type and biogeographic region. Boxplots along each axis capture the distribution of the model errors, while the dashed line indicates a 1 : 1 relationship.

While the global *D* model presented in Eqn (6) was able to produce unbiased estimates of *D* across a wide range of species, climate zones and tree sizes (Fig. 3), scaling relationships between *D* and *H × CD* did vary among both forest types and functional groups (Fig. 4). Incorporating these differences in the modelling processes further improved the precision of *D* estimates (Fig. 5 and Table S2). In particular, accounting for the different scaling relationships of angiosperms and gymnosperms reduced the RMSE of the model to 8.1 cm, the average CV to 35.8% (from 43.3% in the global *D* model), and *σ*_*v*_ to 0.35 (Table S2). These gains in precision were especially evident when attempting to predict *D* for angiosperm trees in boreal and temperate coniferous forests, which tend to be dominated by gymnosperms (Fig. 5b). A full list of group‐, forest type‐ and region‐specific *D* equations is provided in Appendix S4.

### Approach 2: estimating aboveground biomass

**Figure 6:**
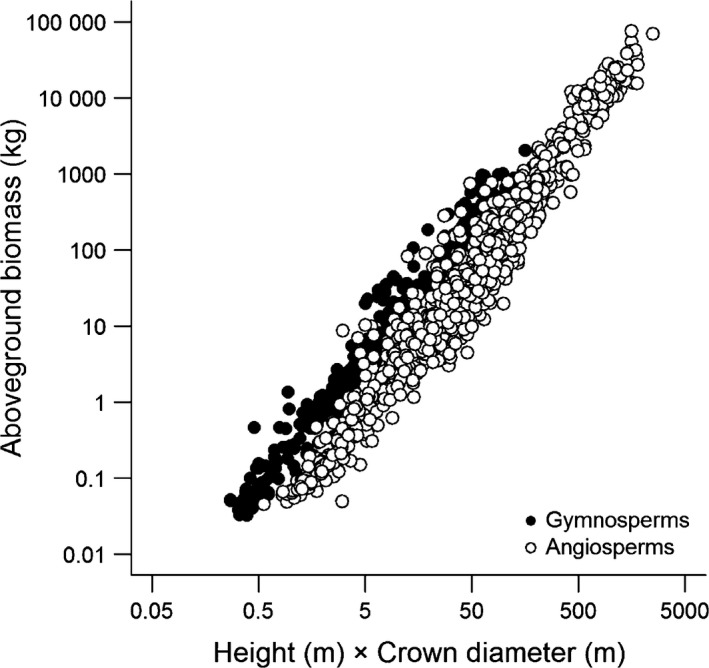
Relationship between aboveground biomass and the product of tree height and crown diameter. Gymnosperm (filled circles; *n *=* *1049) and angiosperm trees (empty circles; *n *=* *1346) are shown separately. For illustrative purposes, 536 trees with a stem diameter of <5 cm are also shown.

*AGB* was strongly related to *H × CD*, with a linear log–log relationship holding across more than six orders of magnitude variation in tree mass (Fig. 6). Scaling relationships between *AGB* and *H × CD* varied consistently among functional groups, with gymnosperms exhibiting higher scaling constants (*α *= 0.109 vs. 0.016) but smaller scaling exponents (*β *= 1.790 vs. 2.013) compared to angiosperm trees (Fig. 6). The best‐fit *AGB* model which accounted for different scaling relationships among angiosperms and gymnosperms was

(7) $${AGB}_{\mathrm{pred}}=\left(0.016+\alpha_{G}\right)\times {\left(H\times CD\right)}^{\left(2.013+\beta_{G}\right)}\times \exp\left[0.204^{2}/2\right]$$

where *α*_*G*_ and *β*_*G*_ are functional group‐dependent parameters which represent the difference in the scaling constant *α* and scaling exponent *β* between angiosperm and gymnosperm trees. For gymnosperms, *α*_*G*_ = 0.093 and *β*_*G*_ = −0.223, whereas for angiosperms both parameters are set to zero. The estimated *σ*_*v*_ of the model was 0.69.

### Comparing approaches 1 and 2

**Figure 7:**
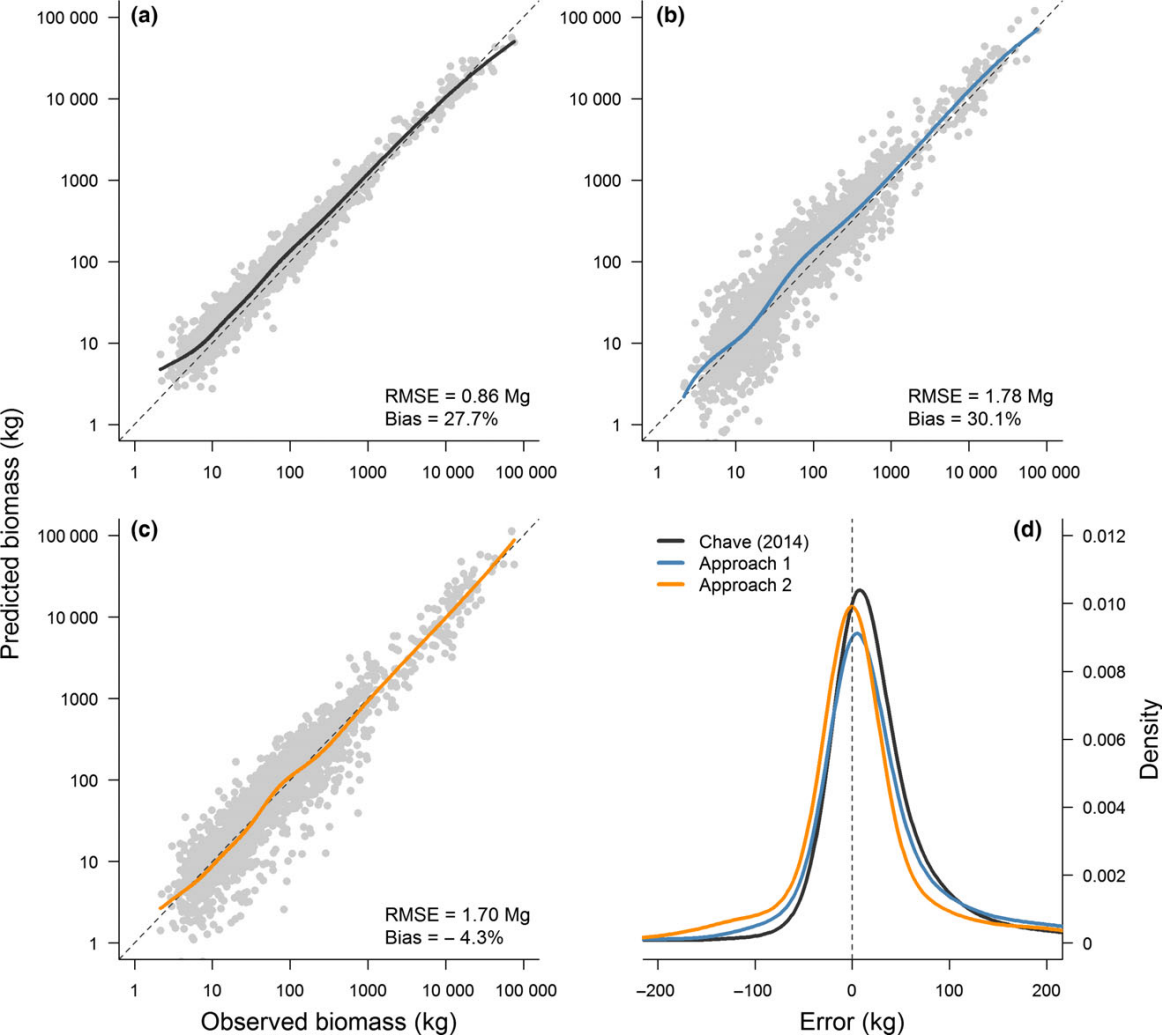
Aboveground biomass (AGB) estimation accuracy. Panels (a–c) show predicted vs. observed AGB values for trees >5 cm in diameter (*n *=* *1859). In panel (a), AGB was estimated using Chave *et al*.'s (2014) equation (where AGB is expressed as a function of diameter, height and wood density). Panel (b) illustrates the predictive accuracy of Chave *et al*.'s (2014) equation when field‐measured diameters are replaced with ones predicted using the global diameter model (i.e. Approach 1). Panel (c) corresponds to a model in which AGB is expressed directly as a function of tree height and crown diameter (i.e. Approach 2). For panels (a–c), the dashed line corresponds to a 1 : 1 relationship, while the solid line is a regression spline fit to the data points to highlight how predictive accuracy varies with tree size. The RMSE and bias of each set of predictions is reported in the lower right‐hand corner. Panel (d) shows the probability density distribution of the absolute errors (i.e. AGB_pred_ – AGB_obs_) for each AGB function.

AGB estimates obtained using Chave *et al*.'s (2014) biomass equation and field‐measured *D* values as inputs showed a close agreement with observed *AGB* values (RMSE = 0.86 Mg; Fig. 7a), but had a tendency to overestimate *AGB* (bias = 27.7%). As expected, replacing field‐measured *D* values with ones predicted using the global *D* model [i.e. Eqn (6), corresponding to Approach 1] increased the RMSE of the model predictions to 1.78 Mg (Fig. 7b). However, the average systematic bias in the *AGB* predictions was little affected (bias = 30.1%, the overestimation arising from the use of the biomass function, not the global *D* model). This suggests that diameter estimates obtained using the global *D* model can be scaled up to biomass without introducing a systematic bias. In contrast to Approach 1, using Eqn (7) to estimate *AGB* directly from *H × CD* (i.e. Approach 2) resulted in substantially lower average bias in *AGB* estimates, regardless of tree mass (bias = −4.3%; Fig. 7c). Furthermore, Approach 2 had the advantage of reducing the RMSE of the model predictions to 1.70 Mg.

## Discussion

We developed general allometric models for estimating both the stem diameter and aboveground biomass of trees based on crown architectural properties which can be remotely sensed: tree height and crown diameter. Here, we discuss how these allometric models can be used to integrate remote sensing imagery – particularly ALS data – into forest monitoring programmes, allowing carbon stocks to be mapped with accuracy across forest landscapes and shedding light on the processes which govern the structure and dynamics of forest ecosystems.

### Stem diameter allometries for remote sensing imagery

We found that estimating stem diameter required accounting for both height and crown size – the latter of which proved essential for differentiating between trees of similar height but having substantially different trunk sizes (King, 2005; King & Clark, 2011). Using a simple metric which combines these two allometric dimensions – *H × CD* – we were able to derive a global equation for estimating stem diameter which proved robust across a large range of tree sizes, forest types and tree species (Fig. 3). Our results highlight how allocation to height growth and lateral crown expansion are strongly coordinated in trees (Sterck & Bongers, 2001; King, 2005; Iida *et al*., 2012) and illustrate how these developmental constraints can be exploited for the purposes of estimating stem diameter.

While we did find that a single allometric function can be used to estimate diameter without introducing systematic bias, incorporating different scaling relationships among forest types, biogeographic regions and functional groups into the models helped improve the predictive accuracy of the allometric equations (Figs 4 and 5; Table S2). Particularly important in this respect was accounting for differences between angiosperms and gymnosperms (Fig. 5b). This is not surprising given the contrasting crown architecture of these two groups: gymnosperms generally exhibit strong apical dominance and invest heavily in height growth, whereas angiosperm trees have a greater ability to plastically adapt the shape and size of their crown to suit their competitive environment (Poorter *et al*., 2012; Hulshof *et al*., 2015). These differences in crown architecture – coupled with clearly distinct leaf biochemical profiles – also mean that angiosperm and gymnosperm trees can be easily distinguished using a variety of remote sensing products (e.g. aerial photographs, hyperspectral sensors and ALS; Dalponte *et al*., 2012). Consequently, we suggest that users select group‐specific diameter equations (which we provide in Appendix S4) wherever possible, as these can be employed with little or no need for additional field data. As our ability to remotely map tree species improves (e.g. through the development of spectral libraries derived from hyperspectral sensors; Asner, 2013), it is conceivable that species‐specific diameter equations could also be utilized in the future. Similarly, other aspects known to influence crown architecture (e.g. tree packing density; Jucker *et al*., 2015) could also be incorporated to further refine the models we develop here.

The diameter allometries we develop here open the door to a more general and robust framework for monitoring forest carbon stocks using ALS. Currently, the standard approach for estimating carbon stocks from ALS data involves calculating summary statistics from ALS point clouds for a given pixel of land (e.g. top canopy height) and relating these to carbon estimates obtained from permanent field plots in a regression framework (Asner & Mascaro, 2014; Asner *et al*., 2014). Despite recent attempts to generalize this ‘area‐based’ approach (e.g. Asner & Mascaro, 2014), most models for estimating carbon stocks from ALS summary statistics are highly site‐specific and can only be applied with confidence to the particular patch of forest they were calibrated for. Working at tree‐level provides an intuitive solution to the issue of developing a general approach for mapping forest carbon stocks and would allow a direct comparison to field‐based aboveground carbon estimates. This ‘tree‐centric’ approach is not without its limitations, the biggest of which is the implicit assumption that individual trees can be reliably identified and measured from ALS point clouds (something which can be challenging in dense, multilayered canopies). However, recent years have seen substantial progress in this respect, as both ALS instruments and the algorithms used to delineate trees from ALS data have improved considerably (Popescu *et al*., 2003; Yao *et al*., 2012; Duncanson *et al*., 2014; Paris *et al*., 2016; Shendryk *et al*., 2016). For example, Paris *et al*. (2016) recently developed a segmentation method which was able to correctly delineate the crowns of 97% and 77% of canopy dominant and understorey trees, respectively, as well as accurately measuring the crown dimensions of all segmented trees. Equally promising is Shendryk *et al*.'s (2016) algorithm which segments trees from the bottom up (mimicking the approach used to process terrestrial laser scanning data; Calders *et al*., 2015). As ALS technology continues to improve, ‘tree‐centric’ carbon monitoring programmes are becoming not only feasible, but oftentimes preferable to traditional ‘area‐based’ approaches (Duncanson *et al*., 2015; Dalponte & Coomes, 2016).

In addition to mapping carbon stocks, characterizing the relationships between stem diameter and crown dimensions also has important implications for advancing our understanding of forest dynamics. The most obvious application of the diameter allometries developed here is for characterizing tree size distributions from airborne imagery, something which has proved challenging using traditional ‘area‐based’ approaches (Maltamo & Gobakken, 2014). Tree size distributions are an emergent property of forest ecosystems – arising from demographic processes and competition for space among individual trees (Enquist *et al*., 2009; Kohyama *et al*., 2015) – and are of key interest for understanding forest dynamics, structure and responses to disturbance (Coomes *et al*., 2003; Enquist *et al*., 2009). Intriguingly, recent work suggests that scaling relationships between diameter and crown size govern how trees utilize canopy space and compete for light, thereby having a direct influence on tree size distributions (Taubert *et al*., 2015; Farrior *et al*., 2016). ALS data, coupled with allometric equations for converting crown dimensions to diameter distributions, would allow us to empirically test this theory across large spatial scales and diverse forest types. In a similar vein, diameter allometries provide a simple solution for integrating ALS data into individual‐based models of forest dynamics (e.g. Shugart *et al*., 2015), allowing these models to be more easily parameterized and validated.

### Estimating aboveground biomass from crown dimensions

Using the subset of trees that were destructively harvested and weighed, we showed that AGB was strongly related to tree height and crown size (Fig. 6). These results give weight to recent reports which have highlighted how accounting for crown size can substantially improve AGB estimation, especially in the case of large trees where a considerable proportion of the biomass is stored in large branches (Henry *et al*., 2010; Goodman *et al*., 2014; Ploton *et al*., 2016). The strong link between crown dimensions and AGB has important implications for ‘tree‐centric’ carbon mapping approaches, as it suggests that AGB can be estimated directly from remotely sensed measurements of tree height and crown width without needing to first predict diameter (Fig. 7c). This is particularly appealing as it reduces the number of steps in the AGB estimation process (each of which carries a certain degree of error) and also eliminates the need to select an equation from the literature for scaling from diameter to AGB.

Our analysis revealed clear differences in the AGB scaling relationships of angiosperms and gymnosperms (Fig. 6), presumably reflecting differences in both crown architecture and wood density among these two groups (Chave *et al*., 2009; Poorter *et al*., 2012; Hulshof *et al*., 2015). It may well be that AGB scaling relationships also vary systematically among forest types or biogeographic regions and that accounting for these differences could further improve the predictive accuracy of the biomass allometries presented here. Unfortunately, the relatively modest sample size of trees with measured AGB at our disposal meant we were unable to robustly test these assumptions. Despite recent efforts to compile comprehensive allometric databases (e.g. Chave *et al*., 2014; Falster *et al*., 2015), the number of trees with measured AGB remains relatively small, geographically biased and heavily skewed towards smaller stems. This is even more so when attempting to find trees that have been felled and weighed and whose crown dimensions have also been recorded. Future studies developing AGB equations should take care to also record the crown dimensions of harvested trees (e.g. Henry *et al*., 2010; Goodman *et al*., 2014; Ploton *et al*., 2016). In this regard, perhaps the most promising solution for bolstering existing allometric databases is terrestrial laser scanning, which captures tree architecture in exquisite detail and provides a nondestructive method for accurately estimating AGB (Calders *et al*., 2015). Most importantly, this would provide access to biomass data for large trees (e.g. ≥10 Mg), which tend to be disproportionately rare in allometric databases – including the one we have assembled here (only 2.4% of measured trees had a mass ≥10 Mg; see Fig. 2c).

### Seeing the forest and the trees

Accurate assessments of forest carbon stocks are essential for initiatives to mitigate climate change – such as the UN's programme for reducing emissions from deforestation and forest degradation (REDD+) – to be implemented successfully (Agrawal *et al*., 2011). Yet monitoring carbon stocks across large and sometimes remote areas of forest poses a real challenge, particularly in countries where national‐scale forest inventory programmes are not in place. In this context, remote sensing technologies such as ALS promise to revolutionize the way we census forests (Asner *et al*., 2014). It is our hope that the allometric equations developed here can help us move towards a more general and robust approach for monitoring forests from the air.

## Statement of authorship

T.J. and D.A.C. designed the study, with the assistance of J.P.C. and J.C.; T.J. and J.P.C. compiled the database, with all authors providing data; T.J. analysed the data and wrote the first draft of the manuscript, with all authors contributing substantially to revisions.

## Supporting information


**Appendix S1.** Database generation
**Table S1.** List of data sources included in the database
**Figure S1.** Height – stem diameter ratio and crown diameter – stem diameter ratio as a function of tree size for each forest type.
**Appendix S2.** Data binning
**Figure S2.** Relative error as a function of tree size for different modelling approaches used to relate stem diameter to the product of tree height and crown diameter
**Figure S3.** Prediction intervals for the relationship between stem diameter and the product of tree height and crown diameter.
**Appendix S3.** Diameter model comparison
**Table S2.** Comparison of the predictive accuracy of allometric models.
**Figure S4.** Relative error as a function of tree size for different diameter models.
**Appendix S4.** Region‐, forest type‐ and group‐specific diameter equations
**Figure S5.** Scaling relationships between height and stem diameter and crown diameter and stem diameter for angiosperm and gymnosperm trees.
**Figure S6.** Effects of mean annual precipitation and temperature on allometric scaling relationships.
**Table S3.** Regional stem diameter allometries
**Table S4.** Regional stem diameter allometries for angiosperms and gymnosperms

**Appendix S5.** R code for implementing data binning approach
