# Supplementary material for: Allometric equations for integrating remote sensing imagery into forest monitoring programmes
Source: Glob Chang Biol. 2016 Jul 6;23(1):177–90. doi: 10.1111/gcb.13388 (PMC6849852; doi:10.1111/gcb.13388)
Supplement: Supplementary file 1 — Appendix S1. Database generation Table S1. List of data sources included in the database Figure S1. Height – stem diameter ratio and crown diameter – stem diameter ratio as a function of tree size for each forest type. Appendix S2. Data binning Figure S2. Relative error as a function of tree size for different modelling approaches used to relate stem diameter to the product of tree height and crown diameter Figure S3. Prediction intervals for the relationship between stem diameter and the product of tree height and crown diameter. Appendix S3. Diameter model comparison Table S2. Comparison of the predictive accuracy of allometric models. Figure S4. Relative error as a function of tree size for different diameter models. Appendix S4. Region‐, forest type‐ and group‐specific diameter equations Figure S5. Scaling relationships between height and stem diameter and crown diameter and stem diameter for angiosperm and gymnosperm trees. Figure S6. Effects of mean annual precipitation and temperature on allometric scaling relationships. Table S3. Regional stem diameter allometries Table S4. Regional stem diameter allometries for angiosperms and gymnosperms [file GCB-23-177-s001.docx]

**Supporting information**

[Appendix S1 | Database generation 2](#_Toc452798010)

[Measurement protocols 4](#_Toc452798011)

[Data screening 5](#_Toc452798012)

[Appendix S2 | Data binning 7](#_Toc452798013)

[Appendix S3 | Diameter model comparison 12](#_Toc452798014)

[Appendix S4 | Region-, forest type- and group-specific diameter equations 13](#_Toc452798015)

[Region- and forest type-specific equations 15](#_Toc452798016)

[Functional group-specific equations for each forest type and biogeographic region 16](#_Toc452798017)

[References 17](#_Toc452798018)

## Appendix S1 | Database generation

**Table S1 |** List of data sources included in the database.

| **Region** | **Country** | **Data source** | **Reference** |
| --- | --- | --- | --- |
| Global | Multiple countries | Open access database | Falster *et al.* (2015) |
| Global | Multiple countries | Journal article | Chave *et al.* (2014) |
| Global | Multiple countries | Open access database | http://elar.usfeu.ru/handle/123456789/4931?locale=en |
| Global | Multiple countries | Journal article | Moncrieff *et al.* (2014) |
| Africa | Multiple countries | Institutional database | http://vmamapgn-test.mpl.ird.fr:8080/geonetwork/srv/eng/search#\|7dd46c7d-db2f-4bb0-920a-8afe4832f1b3 |
| Africa | Multiple countries | Journal article | Ploton *et al.* (2016) |
| Africa | Ghana | Journal article | Henry *et al.* (2010) |
| Africa | Liberia | Journal article | Poorter *et al.* (2003) |
| Asia | China | Journal article | Guisasola *et al.* (2015) |
| Asia | China | Unpublished data | Xiang *et al*. (unpublished) |
| Asia | India | Journal article | Antin *et al.* (2013) |
| Asia | Indonesia | Journal article | Schlund *et al.* (2015) |
| Asia | Malaysia | Journal article | Sterck *et al.* (2001) |
| Asia | Malaysia | Journal article | Iida *et al.* (2012) |
| Europe | Multiple countries | Institutional database | [http://icp-forests.net](http://icp-forests.net/)**^1^**  Dobbertin *et al.* (2013) |
| Europe | Multiple countries | Journal article | Jucker *et al.* (2015) |
| Europe | Multiple countries | Journal article | Wirth *et al.* (2004) |
| Europe | Switzerland | Institutional database | http://www.wsl.ch/info/organisation/fpo/lwf/index_EN |
| Europe | Italy | Journal article | Dalponte & Coomes (2016) |
| North America | Canada | Unpublished data | Marshall *et al*. (unpublished) |
| North America | Canada | Journal article | Caspersen *et al.* (2011) |
| North America | Canada | Journal article | Groot & Luther (2015) |
| North America | Canada | Institutional database | https://www.agric.gov.ab.ca/app21/forestrypage |
| North America | Mexico | Journal article | Bongers *et al.* (1988) |
| North America | USA | Open access database | <http://www.fia.fs.fed.us/tools-data/other_data/index.php> |
| North America | USA | Open access database | Anderson-Teixeira *et al.* (2015) |
| North America | USA | Journal article | Cole & Lorimer (1994) |
| Oceania | New Zealand | Journal article | Coomes *et al.* (2014) |
| South America | Brazil | Open access database | http://mapas.cnpm.embrapa.br/paisagenssustentaveis http://dx.doi.org/10.3334/ORNLDAAC/1301 |
| South America | Brazil | Open access database | http://dx.doi.org/10.5061/dryad.p281g  Goodman *et al.* (2014) |
| South America | Bolivia | Journal article | Poorter *et al.* (2006) |

**^1^**Data from the ICP Forests Level II plot network for the following countries were included in the database: France, Belgium, Germany, UK, Portugal, Spain, Luxembourg, Finland, Hungary, Poland, Latvia and Serbia.

### Measurement protocols

Due to the geographic scope of the database and the fact that the studies from which we obtained data were conducted over the span of several decades, the protocols used to measure the stem diameter (*D*), height (*H*), crown diameter (*CD*) and aboveground biomass (*AGB*) of trees differ to some extent among data sources. Here we outline the approaches used to measure each of these attributes:

**Stem diameter**: *D* is typically recorded at a height of 1.3 m off the ground using a tape measure, or alternatively with the aid of a caliper for small stems. Note that in the case of buttressed trees, the standard practise is to measure *D* above buttress (although for data obtained from compiled databases it is impossible to determine whether this convention was followed). All *D* measurements were rounded to the nearest 0.1 cm.

**Tree height**: with the exception of small trees whose height could be measured directly from the ground and a small number of trees that were climbed, in the majority of cases *H* was measured using a vertex hypsometer or laser range finder (“sine” method *sensu* Larjavaara & Muller-Landau, 2013). Alternatively, some studies employed a clinometer to measure tree height (“tangent” method *sensu* Larjavaara & Muller-Landau, 2013). Measuring *H* in dense closed-canopy forests – where tree tops are hard to identify clearly – using either of these two approaches can be challenging (Larjavaara & Muller-Landau, 2013). Of the two, *H* estimates obtained using the “tangent” method have been shown to be less precise, although vertex hypsometers and laser range finders may in some cases systematically underestimate *H* (Larjavaara & Muller-Landau, 2013). All *H* measurements were rounded to the nearest 0.1 m.

**Crown diameter**: different attributes of crown size were reported across studies, including diameter, radius and area. In all cases, crown dimensions reflect the maximum extension of the crown, and were calculated as the average value of multiple crown measurements taken in the field using either a tape measure or a laser range finder (2 – 8 measurements per tree). All crown size measurements were converted to *CD*, and were rounded to the nearest 0.1 m.

**Aboveground biomass**: to measure *AGB*, trees were first felled at ground level and divided into sections (which included the main stem, branches and, where possible, leaves). These sections were then weighed fresh, and their oven-dry weight (in kg) was determined by subtracting the measured water content of the different plant organs. For sections that were too large to be weighed directly, wood volume was first calculated based on geometric measurements (i.e., length, circumference and taper of the tree section). Oven-dry weight was then estimated by multiplying the wood volume by the wood’s specific gravity (Chave *et al.*, 2014). All *AGB* measurements were rounded to the nearest 0.1 kg.

### Data screening

Outliers – defined as trees that exhibit *H–D* or *CD–D* scaling relationships which depart strongly from the mean – can disproportionately influence parameter estimates in a regression framework. Outliers can result from gross data entry errors (e.g., reporting centimetres as meters or *vice versa*), or alternatively may indicate the presence of a damaged tree (e.g., broken main stem or major branch). Visual inspection of the data revealed no obvious gross data entry errors. However, a certain number of trees were either (i) particularly short or (ii) had very small crowns for a given stem diameter, suggesting they may be damaged. For data sources which reported tree damage in their surveys, we used available records to filter out damaged trees from the database. For surveys which did not report tree damage, we developed an approach based on quantile regression to identify potentially damaged trees based on their *H–D* and *CD–D* scaling relationships. Using height–diameter scaling as an example, we first calculated the ratio between height and diameter (*H*/*D*) for each tree. The *H/D* ratio tends to decrease with increasing tree size (see Fig. S1). To identify trees with unusually small *H/D* ratios for a given stem size (which would suggest damage to the main stem), we therefore used quantile regression to relate *H/D* ratios to stem diameter on a log-log scale. The regression was fit to the lower 0.1% of the data, and tree’s falling below the regression line were assumed to be severely damaged and were dropped from the database (Fig. S1). This same process was employed to identify trees with unusually small *CD–D* ratios (indicating either physical damage to major branches or crown dieback as a result of drought, insect damage and/or old age), and was undertaken separately for each forest type (as *H/D* and *CD/D* ratios vary among forest types; see Fig. S1). In total, 218 trees were removed from the database, corresponding to 0.2% of the entire dataset.


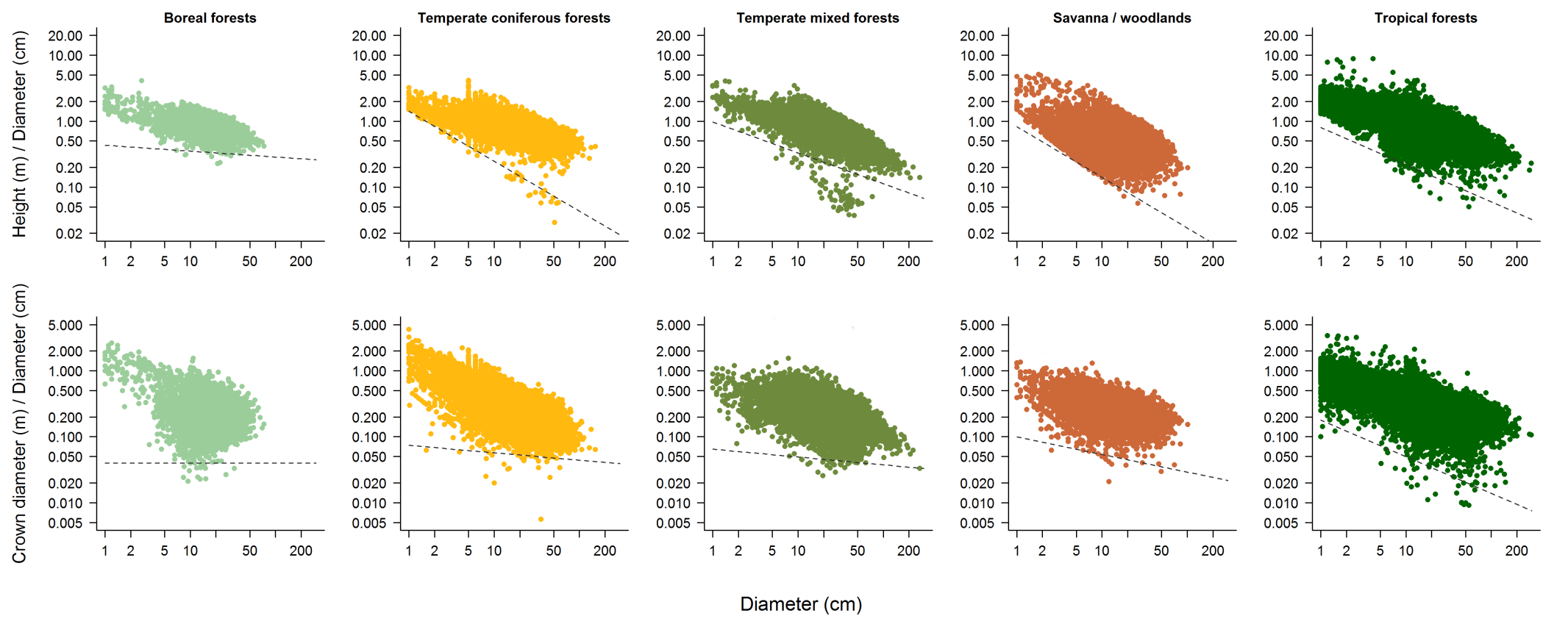


**Fig. S1 |** Height – stem diameter ratio and crown diameter – stem diameter ratio as a function of tree size for each forest type. Dashed lines are quantile regressions fit to the 0.1 percentile of the data. Points falling below these lines correspond to trees that are either unusually short (top row) or have particularly small crown diameters (bottom row) for a given stem diameter, suggesting they may have been damaged (e.g., broken stems or major branches). We therefore removed these trees the database (*n* = 218).

## Appendix S2 | Data binning

Allometric equations are generally derived by fitting ordinary least squares (OLS) linear regressions directly to field data (which in most cases have been log-transformed). However, in some cases this approach can result in severely biased parameter estimates, making it impossible to use the equations to derive reliable estimates of the response variable. There are several reasons why OLS regression can produce systematically biased estimates. For instance, OLS regression will tend to underestimate bivariate slopes when the independent variable in a model is measured with error [in our case height (*H*) and crown diameter (*CD*)], an issue which is commonly referred to as the “regression dilution bias” (Fuller, 1987; Frost & Thompson, 2000). This issue is exacerbated by the tendency of allometric data to exhibit non-constant variance (i.e., increasing variability for higher mean values) and a strongly skewed distribution (i.e., the fact that small stems vastly outnumber large ones).

Using stem diameter (*D*) as an example, a preliminary analysis of our data revealed that using OLS regression to model the relationship between *D* and *H × D* (on a log-log scale) yielded systematically biased estimates of *D* (see green lines in Fig. S2). Specifically, the model tended to severely overestimate *D* for small trees, while concurrently overestimating that of large ones. Several approaches have been put forward to deal with the regression dilution bias. Here we compare four alternative methods: (i) standardized major axis (SMA) regression, (ii) weighted regression, (iii) data binning and (iv) data thinning.

1. *SMA regression*: in contrast to OLS regression – where the objective is to minimise the sum of squares of the residuals measured on the *y* axis (i.e., the response variable) – in model II regression approaches such as SMA the line of best fit is obtained by minimizing the distance to the data along both the *x* and *y* axes (Warton *et al.*, 2006). SMA regression is better suited than OLS for estimating the true slope of a bivariate line, but has the drawback of not being designed as a predictive tool. SMA regression was implemented using the *sma* function in R (Warton *et al.*, 2012).
2. *Weighted regression*: whereas OLS regression assumes constant variance in the residuals, weighted regression accounts for the tendency of the residual variance to increase with the mean of the fitted values (a common pattern in allometric data). This is achieved by associating each observation with a weight which is inversely proportional to the residual variance (Picard *et al.*, 2012). We fit the weighted model using the *nls* function in R, where *D* was expressed as power-law function of *H × D* [analogous to fitting log(*D*) *vs* log(*H × D*) in a linear modelling framework] and a weighting of 1/*D* was applied.
3. *Data binning*: data binning consists in dividing the data into size classes (in this case 50 stem diameter logarithmic bins of constant width), and then calculating mean values for each allometric variable within these bins (i.e., mean *H*, *CD* and *H × CD* values for each diameter class). Regressions are then fitted to the binned data as opposed to the raw values. As discussed in the main text, the advantage of this approach is that it reduces the among-tree variability in allometric measurements to a mean for each size class, thereby addressing the issue of the regression dilution bias (Duncanson *et al.*, 2015). However, this feature is also the biggest drawback of the binning method, as it inevitably results in an underestimation of the residuals standard deviation (*σ*) of the model. Consequently, any attempt to propagate uncertainty in the model predictions will severely underestimate the actual variability among trees.
4. *Data thinning*: in an attempt to incorporate model uncertainty in our estimation workflow, we tested whether a data thinning approach could be used instead of binning the data. This consisted in first dividing trees into 100 log-diameter classes (as was done for the data binning method), and then selecting an equal number of trees – 100 chosen at random – in each diameter class. Regressions were then fit to the thinned data to estimate model parameters.

We compared the performance of each of the above approaches using the workflow described in the main text. Specifically, we (i) divided the database into a training set (90% of the data) and a validation set (remaining 10% of the data selected at random following a size-stratified random sampling approach). We then (ii) used the training dataset to model the relationship between *D* and *H × D* using each of the four modelling approaches, following which (iii) the models were used to predict *D* for all trees in the validation dataset. To test for systematic bias in the model predictions, we then (iv) calculated the relative errors in the predictions generated from each modelling approach as follows: $\frac{D_{pred}-D_{obs}}{D_{obs}}\times100$. Steps (i-iv) were repeated 100 times to avoid the randomization procedure in step (i) having an undue effect on the model evaluation process.

All four of the above modelling approaches reduced the systematic bias in the model predictions compared to OLS regression (Fig. S2). However, of these only data binning was successful in removing systematic bias altogether. SMA, weighted regression and data thinning all underestimated the true scaling coefficient (i.e., slope) of the *D* *vs* *H × D* relationship. As a result, all three approaches systematically overestimated *D* for small trees, and instead tended to overestimate that of large ones (Fig. S2).


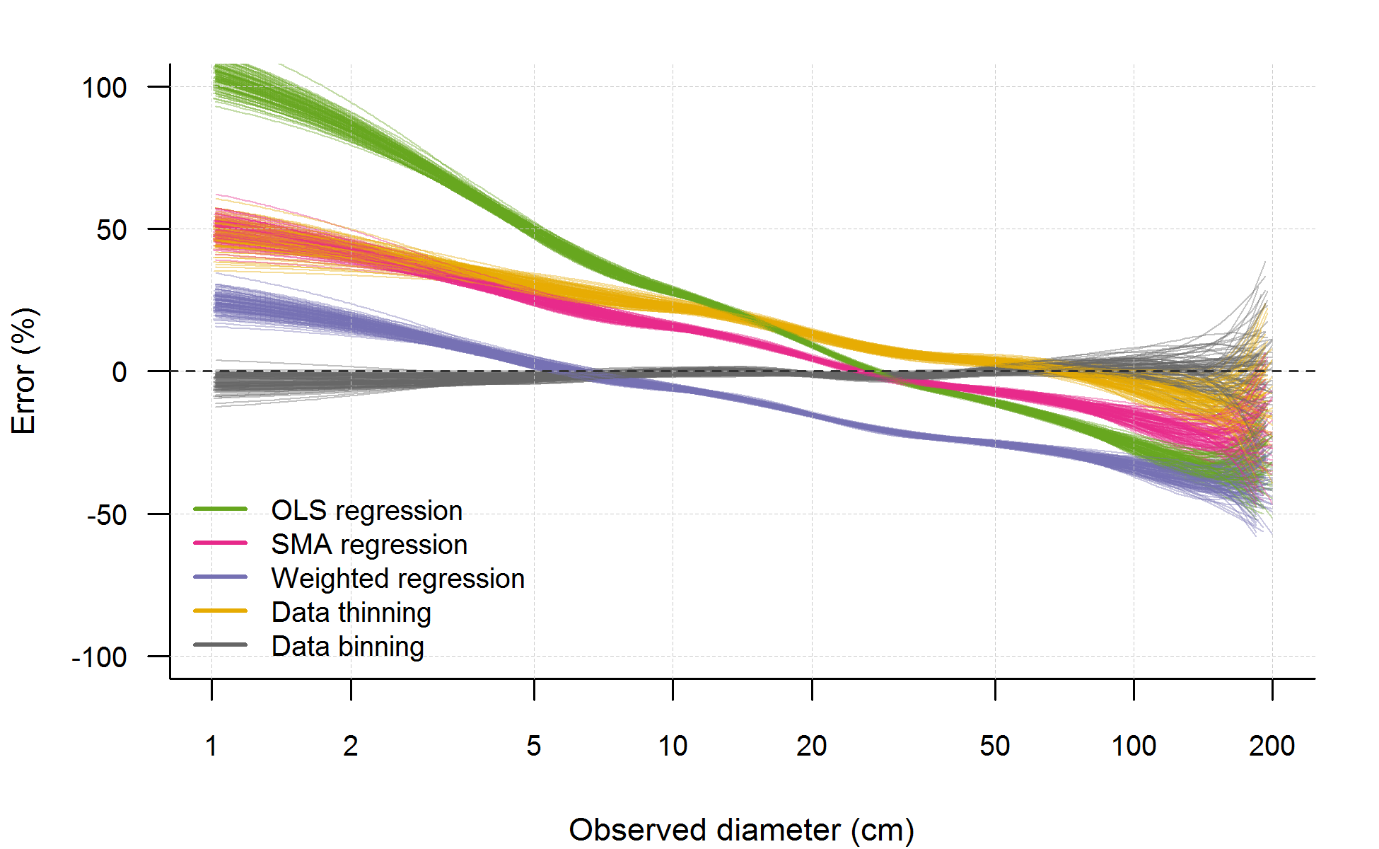


**Fig. S2 |** Relative error as a function of tree size for different modelling approaches used to relate stem diameter to the product of tree height and crown diameter. Relative errors were calculated for a random subset of trees corresponding to 10% of the entire database (*n* = 10875) which were used exclusively to validate the models. This randomization procedure was repeated 100 times, and at each iteration regression splines were used to illustrate how the magnitude of the relative errors varies as a function of tree size for each modelling approach.

Based on the above results, we chose to use the data binning approach for modelling allometric relationships, as it proved the best suited for obtaining unbiased estimates of the response variable. However, to address the issue of model uncertainty (i.e., the underestimation of *σ*) we provide a simple method for capturing the true variability in allometric scaling relationships among individual trees. The method is based on the comparison of predicted and observed values generated using an independent validation dataset. Here we describe the rationale behind the approach and illustrate how it can be used to robustly propagate the uncertainty in the model predictions. Appendix S5 provides R code for implementing this analysis.

In the context of regression, prediction intervals are an estimate of the interval within which future observations are expected to fall, with a certain probability. For a given value of the independent variable *x*, the 95% prediction interval for the predicted values of the response variable *y* ($\hat{y}$) can be calculated as:

| $\hat{y}\pm t^{*}\times\sigma\sqrt{1+\frac{1}{n}+\frac{\left( x-\bar{x} \right)^{2}}{(n-1)\times\sigma_{x}^{2}}}$ |  | (S1) |
| --- | --- | --- |

where *t^*^* is the critical value of the Student’s *t* distribution (which will depend on the desired significance level and on the degrees of freedom), *n* is the number of observation, $\bar{x}$is the mean value of *x*, *σ_x_* is standard deviation of *x*, and *σ* is the standard deviation of the residuals which is given by:

| $\sigma=\sqrt{\frac{\sum\left( y_{i}-\hat{y}_{i} \right)^{2}}{n-2}}$ |  | (S2) |
| --- | --- | --- |

In the case of the data binning approach, the issue with obtaining realistic prediction intervals is that *σ* will almost inevitably be severely underestimated – thereby resulting in overly narrow prediction intervals (see dotted black lines in Fig. S3). This is because the difference between observed and fitted values (i.e., the residuals; $y_{i}-\hat{y}_{i}$) is being calculated not for individual trees, but for mean binned values (which inevitably mask tree-level variation in allometric parameters). However, taking advantage of the independent dataset (the 10% of trees set aside for model validation), we can compare predicted and observed value of *D* generated for individual trees to get a much more realistic estimate of the true value of *σ* (which we refer to as *σ_v_*):

| $\sigma_{v}=\sqrt{\frac{\sum\left( ln\left( D_{obs} \right)-ln\left( D_{pred} \right) \right)^{2}}{n-2}}$ |  | (S3) |
| --- | --- | --- |

Using this simple approach we were able to generate realistic prediction intervals for models fit using the data binning approach (see dashed blue lines in Fig. S3).


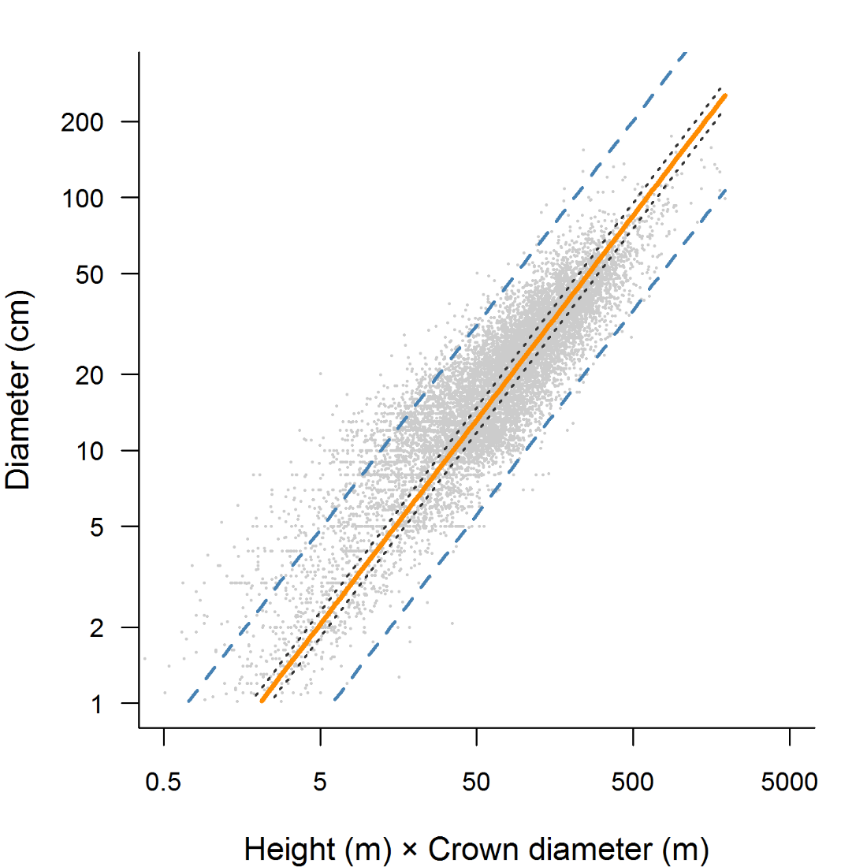


**Fig. S3 |** Prediction intervals for the relationship between stem diameter and the product of tree height and crown diameter. Grey points are individual trees (*n* = 10875; corresponding to 10% of the database which was used exclusively as a validation dataset). The continuous orange line is the line of best fit obtained using the data binning approach. The dotted black lines show the range of the 95% prediction intervals calculated using the estimated value of *σ* obtained directly from the binned model (*σ* = 0.056 in this case). In contrast, the dashed lines in blue are the 95% prediction intervals obtained when using the independent validation dataset to estimate *σ_v_* as described in equation S3 (*σ_v_* = 0.45 in this case).

## Appendix S3 | Diameter model comparison

**Table S2 |** Comparison of the predictive accuracy of allometric models. For each model, the root mean square error (RMSE), average systematic bias, average tree-level coefficient of variation (CV), coefficient of determination (*R*^2^; calculated as $1-\frac{\sum\left( D_{obs}-D_{pred} \right)^{2}}{\sum\left( D_{obs}-\bar{D_{obs}} \right)^{2}}$), and standard deviation of predicted *vs* observed values (*σ_v_*) were calculated on an independent validation dataset corresponding to 10% of trees selected at random. This randomization procedure was repeated 100 times and for each metric the mean value across the 100 iterations was calculated (for RMSE, bias and CV, the ±95% range is reported in brackets). In Model 5 separate intercepts (*α*) and slopes (*β*) were estimated for each biogeographic region *i* and forest type *j*, while Model 6 further allowed *α* and *β* to vary among the *k* functional groups (i.e., angiosperms and gymnosperms).

| **Model** | **Model structure** | **RMSE (cm)** | **Bias (%)** | **CV (%)** | ***R*^2^** | ***σ_v_*** |
| --- | --- | --- | --- | --- | --- | --- |
| M1 | ln(*D*) = *α + β*ln(*H*) | 13.7 (±0.3) | 24.7 (±1.1) | 60.7 (±1.1) | 0.56 | 0.54 |
| M2 | ln(*D*) = *α + β*ln(*CD*) | 16.6 (±1.0) | -4.5 (±1.4) | 73.8 (±4.2) | 0.31 | 0.71 |
| M3 | ln(*D*) = *α + β*ln(*H*) + *γ*ln(*CD*) | 10.3 (±0.4) | -0.9 (±0.8) | 45.4 (±2.0) | 0.69 | 0.46 |
| M4 | ln(*D*) = *α + β*ln(*H* × *CD*) | 9.7 (±0.4) | -1.2 (±0.9) | 43.3 (±1.3) | 0.70 | 0.45 |
| M5 | ln(*D*) = *α_ij_ + β_ij_*ln(*H* × *CD*) | 8.8 (±0.3) | 0.6 (±0.9) | 39.2 (±1.1) | 0.77 | 0.39 |
| M6 | ln(*D*) = *α_ijk_ + β_ijk_*ln(*H* × *CD*) | 8.1 (±0.3) | 1.1 (±0.7) | 35.8 (±1.2) | 0.81 | 0.35 |


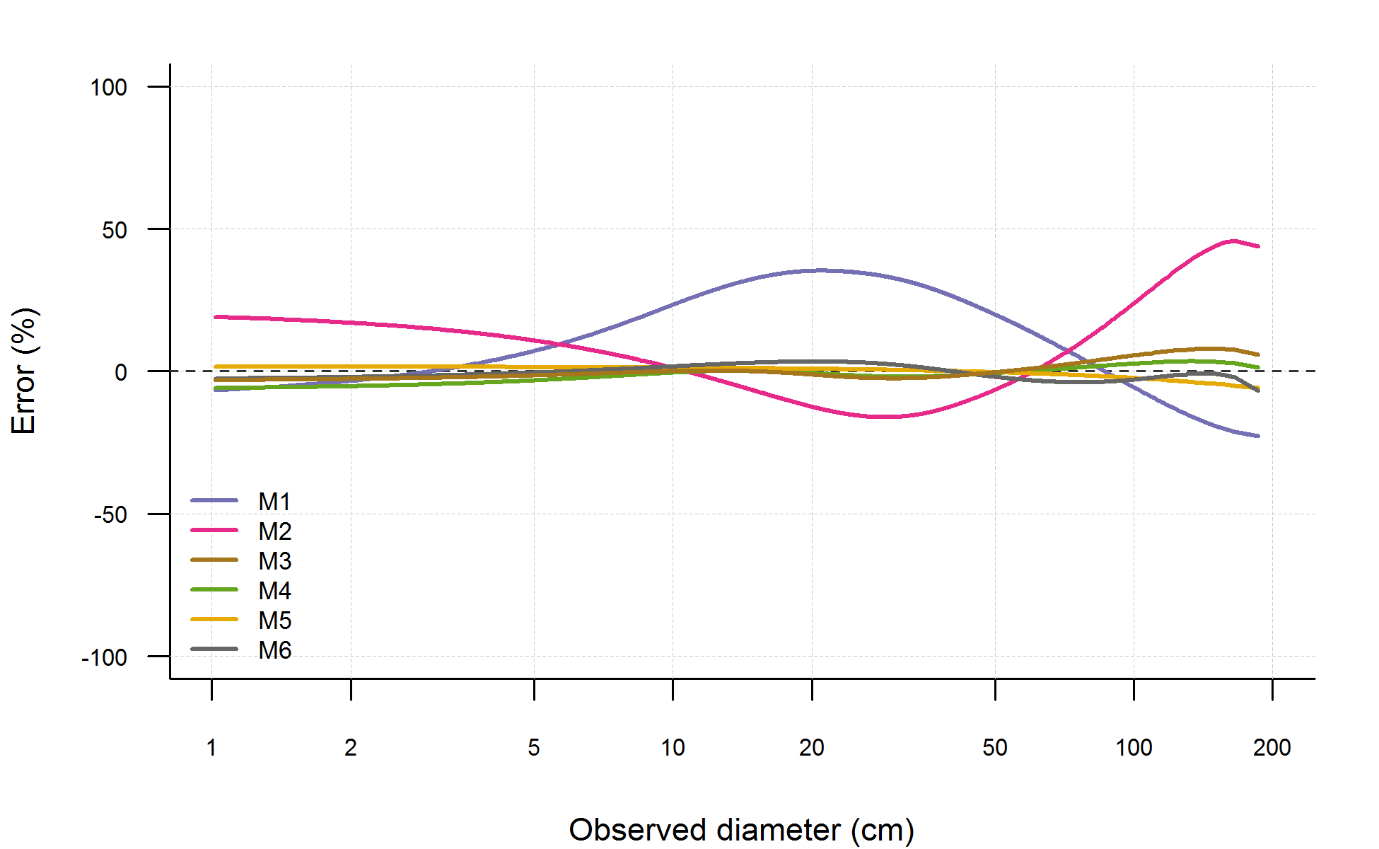


**Fig. S4 |** Relative error as a function of tree size for different diameter models. Relative errors were calculated for a random subset of trees corresponding to 10% of the entire database (*n* = 10875) which were used exclusively to validate the models. Regression splines illustrate how the magnitude of the relative errors varies as a function of tree size for each model.

## Appendix S4 | Region-, forest type- and group-specific diameter equations

Allometric scaling relationships between stem diameter, height and crown diameter vary considerably among forest types, functional groups and geographic regions (see Fig. S5 and Fig. 4 in the main text), with much of this variation arising from differences in climate which determine local species composition and carbon allocation strategies (Lines *et al.* 2012; Hulshof *et al.* 2015; see Fig. S6). Here we provide equations for estimating a tree’s stem diameter based on its height and crown diameter (i.e., *H × CD*) for each region, forest type functional group (Tables S3 and S4).


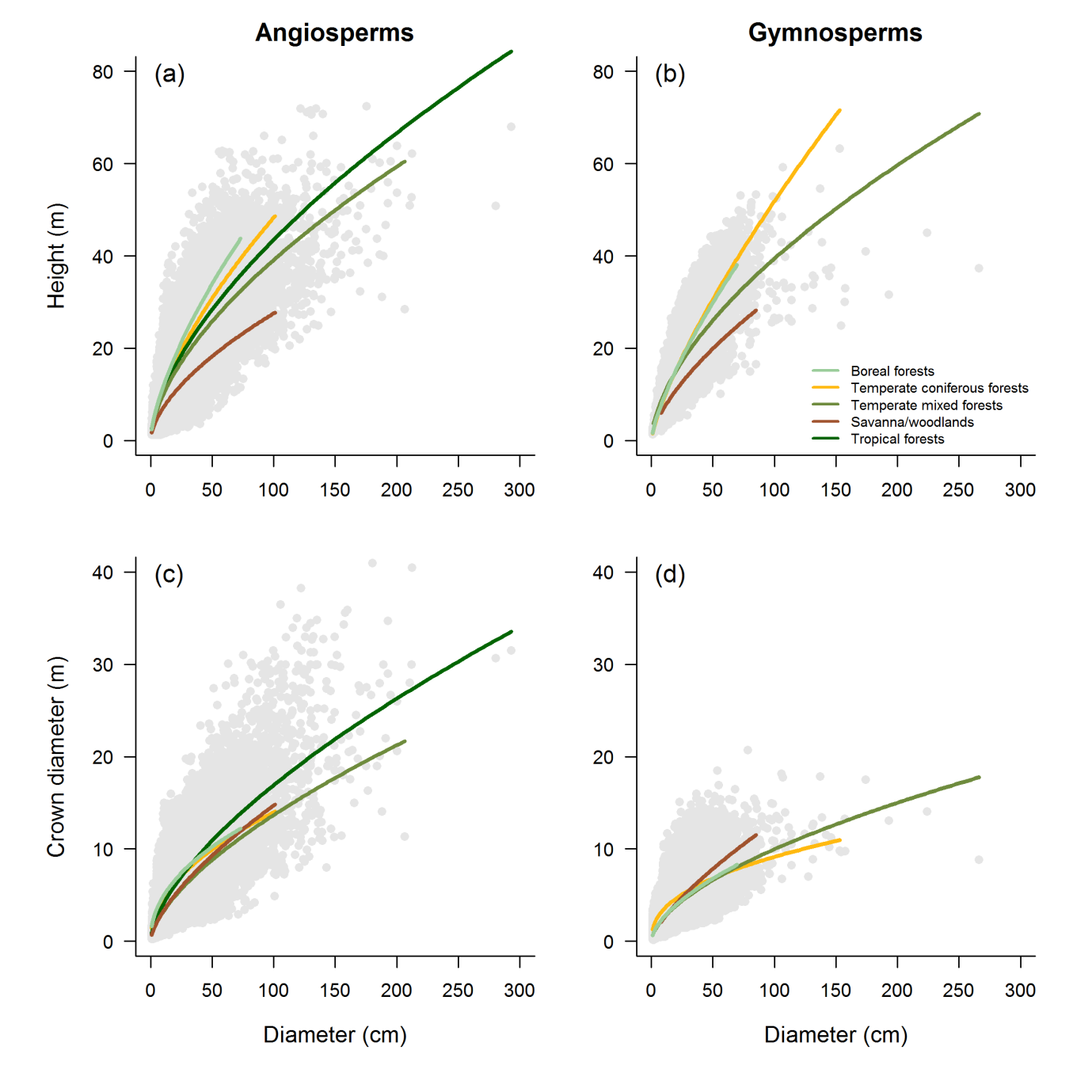


**Fig. S5 |** Scaling relationships between height and stem diameter and crown diameter and stem diameter for angiosperm and gymnosperm trees. Grey points correspond to individual trees, while fitted lines illustrate how scaling relationships vary among forest types.


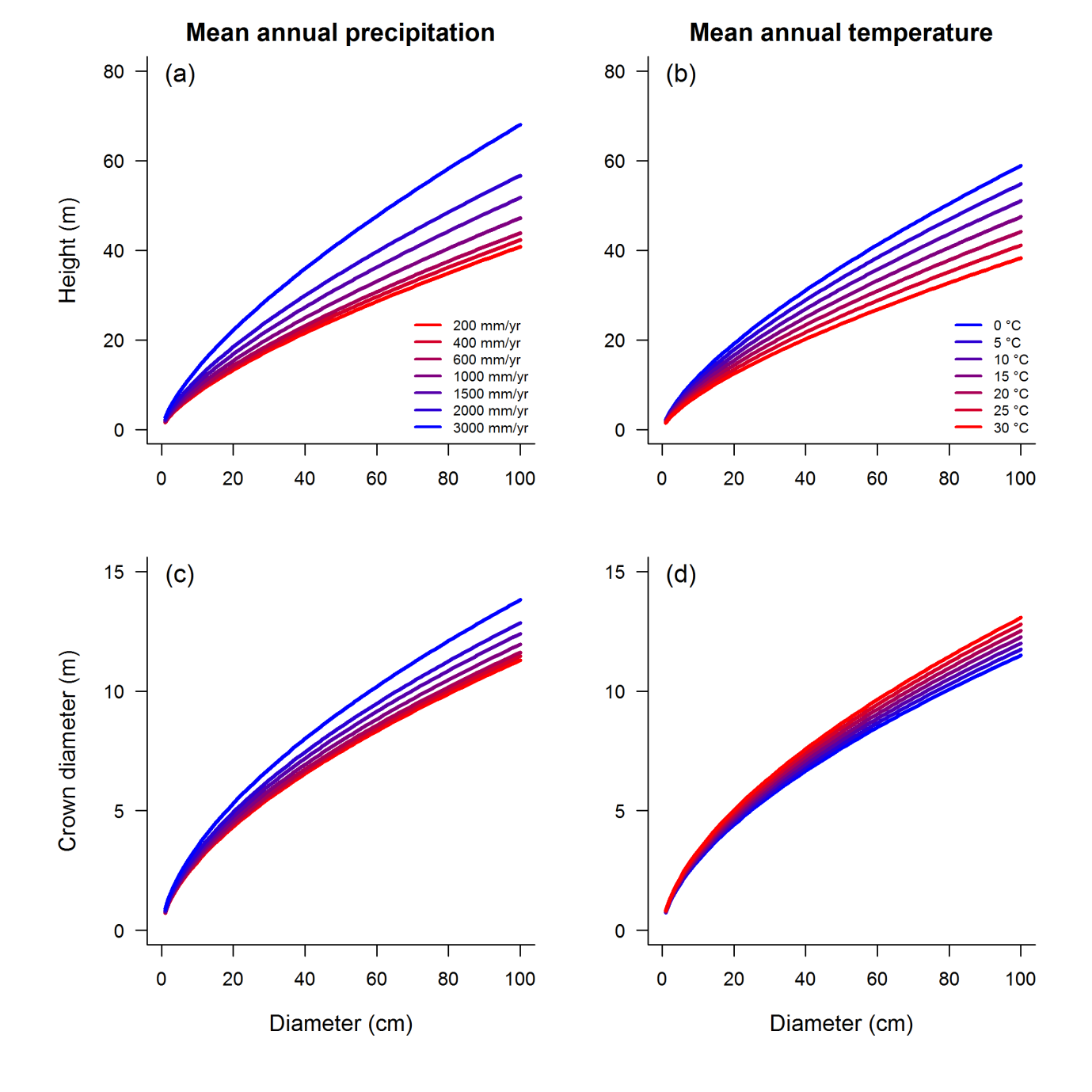


**Fig. S6 |** Effects of mean annual precipitation and temperature on allometric scaling relationships. Curves were obtained from linear models relating *H* and *CD* to *D*, *MAP* and *MAT* [e.g., $\ln\left( H \right)=\beta_{0}+\beta_{1}\ln\left( D \right)+\beta_{2}MAP+\beta_{3}MAT+\varepsilon$], and illustrate how variation in one of the climatic variables [e.g., *MAP* for panels (**a-c**)] influences allometric scaling relationships while keeping the other variable constant at its mean value. For instance, panel (**a**) shows how *H–D* relationships of trees growing at 12.7 °C vary along a MAP gradient ranging from 200 to 3000 mm of rain per year.

### Region- and forest type-specific equations

**Table S3 |** Regional stem diameter allometries. The average tree-level coefficient of variation (CV) is reported for each model subset.

| **Biogeographic region** | **Forest type** | **α** | **β** | **CV (%)** |
| --- | --- | --- | --- | --- |
| Afrotropic | Tropical forests | 0.436 | 0.818 | 45.1 |
| Afrotropic | Woodlands and savannas | 1.267 | 0.749 | 59.4 |
| Australasia | Temperate mixed forests | 0.757 | 0.817 | 39.1 |
| Australasia | Woodlands and savannas | 0.519 | 0.890 | 43.1 |
| Indo-Malaya | Tropical forests | 0.486 | 0.803 | 40.5 |
| Nearctic | Boreal forests | 0.493 | 0.835 | 57.1 |
| Nearctic | Temperate coniferous forests | 0.551 | 0.822 | 40.2 |
| Nearctic | Temperate mixed forests | 0.381 | 0.884 | 32.7 |
| Nearctic | Woodlands and savannas | 0.758 | 0.786 | 33.5 |
| Neotropic | Tropical forests | 0.590 | 0.782 | 41.8 |
| Palearctic | Boreal forests | 1.395 | 0.646 | 26.9 |
| Palearctic | Temperate coniferous forests | 0.331 | 0.941 | 38.9 |
| Palearctic | Temperate mixed forests | 0.708 | 0.753 | 28.5 |
| Palearctic | Tropical forests | 0.537 | 0.793 | 30.6 |
| Palearctic | Woodlands and savannas | 0.819 | 0.785 | 43.4 |

### Functional group-specific equations for each forest type and biogeographic region

**Table S4** Regional stem diameter allometries for angiosperms and gymnosperms. The average tree-level coefficient of variation (CV) is reported for each model subset.

| **Biogeographic region** | **Forest type** | **Functional group** | **α** | **β** | **CV (%)** |
| --- | --- | --- | --- | --- | --- |
| Afrotropic | Tropical forests | Angiosperm | 0.428 | 0.821 | 45.0 |
| Afrotropic | Woodlands and savannas | Angiosperm | 1.268 | 0.750 | 59.6 |
| Australasia | Temperate mixed forests | Angiosperm | 0.769 | 0.811 | 37.9 |
| Australasia | Temperate mixed forests | Gymnosperm | 0.603 | 0.891 | 33.3 |
| Australasia | Woodlands and savannas | Angiosperm | 0.519 | 0.890 | 43.0 |
| Indo-Malaya | Tropical forests | Angiosperm | 0.486 | 0.802 | 40.3 |
| Nearctic | Boreal forests | Angiosperm | 0.286 | 0.865 | 32.9 |
| Nearctic | Boreal forests | Gymnosperm | 0.815 | 0.771 | 49.4 |
| Nearctic | Temperate coniferous forests | Angiosperm | 0.343 | 0.844 | 28.0 |
| Nearctic | Temperate coniferous forests | Gymnosperm | 0.589 | 0.817 | 37.9 |
| Nearctic | Temperate mixed forests | Angiosperm | 0.367 | 0.887 | 31.6 |
| Nearctic | Temperate mixed forests | Gymnosperm | 0.861 | 0.755 | 24.7 |
| Nearctic | Woodlands and savannas | Angiosperm | 0.617 | 0.790 | 25.0 |
| Nearctic | Woodlands and savannas | Gymnosperm | 1.133 | 0.745 | 23.4 |
| Neotropic | Tropical forests | Angiosperm | 0.591 | 0.781 | 41.7 |
| Palearctic | Boreal forests | Angiosperm | 0.467 | 0.839 | 28.1 |
| Palearctic | Boreal forests | Gymnosperm | 1.430 | 0.649 | 23.4 |
| Palearctic | Temperate coniferous forests | Angiosperm | 0.273 | 0.927 | 37.4 |
| Palearctic | Temperate coniferous forests | Gymnosperm | 0.974 | 0.748 | 25.2 |
| Palearctic | Temperate mixed forests | Angiosperm | 0.694 | 0.730 | 23.4 |
| Palearctic | Temperate mixed forests | Gymnosperm | 1.004 | 0.730 | 25.3 |
| Palearctic | Tropical forests | Angiosperm | 0.540 | 0.791 | 30.3 |
| Palearctic | Woodlands and savannas | Angiosperm | 0.910 | 0.738 | 37.9 |
| Palearctic | Woodlands and savannas | Gymnosperm | 1.373 | 0.717 | 26.0 |

## References

Anderson-Teixeira KJ, McGarvey JC, Muller-Landau HC et al. (2015) Size-related scaling of tree form and function in a mixed-age forest. *Functional Ecology*, **29**, 1587–1602.

Antin C, Pélissier R, Vincent G, Couteron P (2013) Crown allometries are less responsive than stem allometry to tree size and habitat variations in an Indian monsoon forest. *Trees - Structure and Function*, **27**, 1485–1495.

Bongers F, Popma J, Meave del Castillo J, Carabias J (1988) Structure and floristic composition of the lowland rain forest of Los Tuxlas, Mexico. *Vegetatio*, **74**, 55–80.

Caspersen JP, Vanderwel MC, Cole WG, Purves DW (2011) How stand productivity results from size- and competition-dependent growth and mortality. *PLoS ONE*, **6**, e28660.

Chave J, Réjou-Méchain M, Búrquez A et al. (2014) Improved allometric models to estimate the aboveground biomass of tropical trees. *Global Change Biology*, **20**, 3177–3190.

Cole WG, Lorimer CG (1994) Predicting tree growth from crown variables in managed northern hardwood stands. *Forest Ecology and Management*, **67**, 159–175.

Coomes DA, Flores O, Holdaway R, Jucker T, Lines ER, Vanderwel MC (2014) Wood production response to climate change will depend critically on forest composition and structure. *Global Change Biology*, **20**, 3632–3645.

Dalponte M, Coomes DA (2016) Tree-centric mapping of forest carbon density from airborne laser scanning and hyperspectral data. *Methods in Ecology and Evolution*.

Dobbertin M, Neumann M, Schroeck H-W (2013) Chapter 10 – Tree Growth Measurements in Long-Term Forest Monitoring in Europe. In: *Developments in Environmental Science*, Vol. 12, pp. 183–204.

Duncanson L, Rourke O, Dubayah R (2015) Small sample sizes yield biased allometric equations in temperate forests. *Scientific Reports*, **5**, 17153.

Falster DS, Duursma RA, Ishihara MI et al. (2015) BAAD: a biomass and allometry database for woody plants. *Ecology*, **96**, 1445.

Frost C, Thompson SG (2000) Correcting for regression dilution bias: comparison of methods for a single predictor variable. *Journal of the Royal Statistical Society: Series A (Statistics in Society)*, **163**, 173–189.

Fuller WA (1987) *Measurement error models* (ed Fuller WA). John Wiley, New York, 440 pp.

Goodman RC, Phillips OL, Baker TR (2014) The importance of crown dimensions to improve tropical tree biomass estimates. *Ecological Applications*, **24**, 680–689.

Groot A, Luther JE (2015) Hierarchical analysis of black spruce and balsam fir wood density in Newfoundland. *Canadian Journal of Forest Research*, **45**, 805–816.

Guisasola R, Tang X, Bauhus J, Forrester DI (2015) Intra- and inter-specific differences in crown architecture in Chinese subtropical mixed-species forests. *Forest Ecology and Management*, **353**, 164–172.

Henry M, Besnard A, Asante WAA et al. (2010) Wood density, phytomass variations within and among trees, and allometric equations in a tropical rainforest of Africa. *Forest Ecology and Management*, **260**, 1375–1388.

Hulshof CM, Swenson NG, Weiser MD (2015) Tree height-diameter allometry across the United States. *Ecology and Evolution*, **5**, 1193–1204.

Iida Y, Poorter L, Sterck FJ, Kassim AR, Kubo T, Potts MD, Kohyama TS (2012) Wood density explains architectural differentiation across 145 co-occurring tropical tree species. *Functional Ecology*, **26**, 274–282.

Jucker T, Bouriaud O, Coomes DA (2015) Crown plasticity enables trees to optimize canopy packing in mixed-species forests. *Functional Ecology*, **29**, 1078–1086.

Larjavaara M, Muller-Landau HC (2013) Measuring tree height: a quantitative comparison of two common field methods in a moist tropical forest. *Methods in Ecology and Evolution*, **4**, 793–801.

Lines ER, Zavala MA, Purves DW, Coomes DA (2012) Predictable changes in aboveground allometry of trees along gradients of temperature, aridity and competition. *Global Ecology and Biogeography*, **21**, 1017–1028.

Moncrieff GR, Lehmann CER, Schnitzler J et al. (2014) Contrasting architecture of key African and Australian savanna tree taxa drives intercontinental structural divergence. *Global Ecology and Biogeography*, **23**, 1235–1244.

Picard N, Saint-André L, Henry M (2012) *Manual for building tree volume and biomass allometric equations: from field measurement to prediction*. FAO, Rome and CIRAD, Montpellier, 215 pp.

Ploton P, Barbier N, Momo ST et al. (2016) Closing a gap in tropical forest biomass estimation: accounting for crown mass variation in pantropical allometries. *Biogeosciences*, **13**, 1571–1585.

Poorter L, Bongers F, Sterck FJ, Wöll H (2003) Architecture of 53 rain forest tree species differing in adult stature and shade tolerance. *Ecology*, **84**, 602–608.

Poorter L, Bongers L, Bongers F (2006) Architecture of 54 moist-forest tree species: traits, trade-offs, and functional groups. *Ecology*, **87**, 1289–1301.

Schlund M, von Poncet F, Kuntz S, Schmullius C, Hoekman DH (2015) TanDEM-X data for aboveground biomass retrieval in a tropical peat swamp forest. *Remote Sensing of Environment*, **158**, 255–266.

Sterck FJ, Sterck FJ, Bongers F, Bongers F, Newbery DM, Newbery DM (2001) Tree architecture in a Bornean lowland rain forest: intraspecic and interspecic patterns. *Plant Ecology*, **153**, 279–292.

Warton DI, Wright IJ, Falster DS, Westoby M (2006) Bivariate line-fitting methods for allometry. *Biological Reviews*, **81**, 259–91.

Warton DI, Duursma RA, Falster DS, Taskinen S (2012) SMATR 3- an R package for estimation and inference about allometric lines. *Methods in Ecology and Evolution*, **3**, 257–259.

Wirth C, Schumacher J, Schulze E-D (2004) Generic biomass functions for Norway spruce in Central Europe - a meta-analysis approach toward prediction and uncertainty estimation. *Tree Physiology*, **24**, 121–39.
